# Invasive mould infections of the central nervous system in the Indian population: a cohort study (2004–2025)

**DOI:** 10.1016/j.lansea.2026.100736

**Published:** 2026-03-04

**Authors:** Harsimran Kaur, Shivaprakash M. Rudramurthy, Rimjhim Kanaujia, Haseen Ahmad, Pravin Salunke, Deepak Bansal, Kirti Gupta, Debajyoti Chatterjee, Chirag Kamal Ahuja, Aastha Takkar Kapila, Manish Modi, Sandeep Mohindra, Rajesh Chhabra, Madhivanan Karthigeyan, Harpreet Singh, Apinderpreet Singh, Ashish Agarwal, Sanjay Verma, Sunil Kumar Gupta, Sanjay Jain, Amita Trehan, Sameer Vyas, Vignesh Pandiarajan, Anup Ghosh, Arunaloke Chakrabarti

**Affiliations:** a Department of Medical Microbiology, Postgraduate Institute of Medical Education and Research (PGIMER), Chandigarh, 160012, India; b Department of Medical Microbiology, Dr. Ram Manohar Lohia Institute of Medical Sciences (RMLIMS), Lucknow, 226021, India; c Department of Neurosurgery, Postgraduate Institute of Medical Education and Research (PGIMER), Chandigarh, 160012, India; d Department of Paediatrics, Postgraduate Institute of Medical Education and Research (PGIMER), Chandigarh, 160012, India; e Department of Histopathology, Postgraduate Institute of Medical Education and Research (PGIMER), Chandigarh, 160012, India; f Department of Radiodiagnosis, Postgraduate Institute of Medical Education and Research (PGIMER), Chandigarh, 160012, India; g Department of Neurology, Postgraduate Institute of Medical Education and Research (PGIMER), Chandigarh, 160012, India; h Department of Internal Medicine, Postgraduate Institute of Medical Education and Research (PGIMER), Chandigarh, 160012, India; i Doodhadhari Burfani Hospital and Research Institute, Haridwar, Uttarakhand, India

**Keywords:** Invasive mould infections, Central nervous system, India, *Aspergillus* spp., *Cladophialophora bantiana*, Brain abscess, Cerebral phaeohyphomycosis

## Abstract

**Background:**

Central nervous system (CNS) invasive mould infection (IMI) is a rare but life-threatening condition. Limited large-scale studies hinder the understanding of its clinical characteristics and optimal management strategies.

**Methods:**

This was a cohort study. We reviewed confirmed patients of CNS IMI (January 2004–March 2025) by microbiological (direct microscopy and/or culture) and/or histopathological evidence at our tertiary care hospital. Clinical, demographic and mycological characteristics were analysed and compared.

**Findings:**

Among 1321 brain abscess/biopsy samples, 127 patients (9.6%) were of fungal origin (adults, 100; paediatrics, 27). The median age was 30 (IQR: 27) years, with male predominance (71.7%). Adults were significantly infected by melanized fungi (p = 0.003). Seventy-four percent of patients had no identifiable underlying immunocompromising condition. The median duration of symptoms was 15 days (IQR: 23 days), including headache (61.4%) and seizures (50.4%). Frequency of fever was significantly higher in infection by melanized fungi (p = 0.02). Frontal lobe involvement was common in paediatric age (OR 0.379, 95% CI 0.141–1.019; p = 0.05). *Aspergillus* spp. (56.3%) and *Cladophialophora bantiana* (21.36%) were the predominant pathogens. *Deniquelata barringtoniae* was reported as a human pathogen. Voriconazole (61.4%) and liposomal amphotericin B (28.3%) were the primary antifungals used. Surgical intervention was performed in all patients. Partial excision (66.7% vs 38.0%, OR 0.306, 95% CI 0.125–0.751; p = 0.01) and use of liposomal amphotericin B (55.6% vs 21%, OR 0.213, 95% CI 0.087–0.522 p = 0.001) were common in paediatric patients. The overall mortality was 30.7%, complete excision was significantly more frequent among survivors than non-survivors (67.0% vs 30.8%; OR 0.218, 95% CI 0.097–0.492; p = 0.001). Lack of headache (p = 0.044) and partial excision surgery (p = 0.001) were independently associated with poor outcome.

**Interpretation:**

We describe a compendium of CNS IMI in patients from India, highlighting distinct clinical patterns and treatment outcomes across age groups and fungal types. This warrants investigation of host and pathogen-related factors in the country.

**Funding:**

Funding included Department of Science and Technology-Science and Engineering Research Board (DST-SERB), New Delhi, India and 10.13039/501100001411Indian Council of Medical Research (ICMR), New Delhi, India.

**Research in context**

**Evidence before this study**

We searched PubMed (1952–January 2026) using “central nervous system (CNS) mycoses”, “CNS invasive mould infections”, “CNS mould infections”, “neurotropic fungi” and additional search terms of “brain abscess”, “meningitis”, “ventriculitis”, “melanized fungi”, “cerebral fungal infections”, “cerebral phaeohyphomycosis”, “cerebral mucormycosis”, “cerebral aspergillosis”, and “*Cladophialophora bantiana*”. CNS invasive mould infections (IMIs) are associated with high mortality worldwide. Epidemiological data on CNS IMIs are sparse with no clear incidence, as most information is limited to case reports and small case series. No comparison has been reported in CNS IMIs in adults or children or those caused by hyaline or melanized fungi. Globally, CNS IMIs account for approximately 1–8% of cerebral abscess patients. Evidence from lower-income and middle-income countries (LMICs) remains even more limited. Anecdotal reports suggest a growing recognition of CNS IMIs in India over the past two decades. However, comprehensive literature detailing risk factors, clinical course, etiological agents and their resistance profiles, treatment modalities, and outcomes is lacking, making it difficult to draw conclusions about their global epidemiology. This study represents a large cohort of CNS IMIs.

**Added value of this study**

The study reports a comprehensive epidemiological data on CNS IMIs from a tropical country. An increasing incidence of CNS IMIs was noted between 2004 and 2025. An overall incidence of 9.6% provides a real picture of CNS IMIs in this region. Among the etiologic agents, six-yearly trend analysis revealed a decline in infections caused by hyaline fungi and a fractional rise in infections by melanized fungi after 2021. More than half of the patients (74%) were immunocompetent. *Aspergillus* species were the most common pathogens (56.3%), with *Aspergillus flavus* predominant in adults (44%) and *Aspergillus fumigatus* in children (42%). *C. bantiana* (21.4%), a neurotropic fungus, predominantly reported from India, was the second most frequent pathogen overall. Mortality was 30.7%. Survivors depicted higher frequency of headache possibly leading to early diagnosis. Adults were more likely to be affected by melanized fungi, while children had a predominance of hyaline fungi. Importantly, we describe *Deniquelata barringtoniae* as a human pathogen. The study is significant in the context of South Asia which has tropical climate conducive to growth of fungi. It highlights the emergence of melanized fungi which are known for thermotolerance possibly adapting well to the global warming and climate change.

**Implications of all the available evidence**

Our findings highlight the rising burden of CNS IMIs in LMICs, particularly affecting young immunocompetent males—likely due to environmental exposure and potentially influenced by climate change and fungal adaptation. The emergence of rare pathogens such as *D. barringtoniae* underscores the evolving landscape of fungal infections. High mortality rates emphasize the need for early clinical suspicion, accurate diagnostic strategies, and combined surgical and antifungal treatment. Increasing awareness of fungal infections in India and other LMICs is essential, as delays in diagnosis and treatment substantially worsen outcomes. Establishing a nationwide CNS IMI registry would facilitate surveillance and research. Given their morbidity and mortality burden, CNS IMIs align with the WHO's recognition of fungal infections as a global health priority and may be considered a candidate for inclusion as a “priority disease” by international health agencies.

## Introduction

Central nervous system (CNS) invasive mould infections (IMIs) affecting both immunocompromised and immunocompetent hosts exhibit exceptionally high mortality and morbidity (60−90%).^1^, ^2^, ^3^, ^4^ The exact burden of CNS IMI is not well defined and is limited to case reports and small case series. Globally, it accounts for ∼1–8% of cerebral abscess patients.^5^ A substantial number of patients have been reported from tropical countries, where the climate is conducive for the proliferation of fungi, like in Asian countries, particularly India. Limited case series from the southern part of India report an incidence ranging from 0.99 to 1.7% over 24–38 years, with mortality rates exceeding 50%, especially in immunosuppressed individuals.^5^, ^6^, ^7^ Despite its clinical significance, there is a lack of literature focusing on the aetiology of CNS IMI.

The rising incidence of fungal infections can be attributed to the burgeoning population of immunocompromised individuals, critically ill patients receiving broad-spectrum antibiotics and prolonged hospitalization. Contributing factors also include the use of newer biological therapies, chemotherapeutic regimens, and infections with emerging pathogens such as the SARS-CoV-2 virus.^8^ CNS IMI generally occurs as a direct invasion/extension of peripheral infections (e.g., mucormycosis) via frontal sinuses or as a part of the hematogenous dissemination leading to brain abscess, and occasionally resulting in cerebral infarction due to septic embolisms, or mycotic aneurysms/ischemia, and/or haemorrhage.^9^ CNS IMIs have been documented in immunocompetent individuals, especially by neurotropic fungi such as *C. bantiana* or *Rhinocladiella mackenziei*.^2^^,^^10^ The diagnosis of CNS IMI is challenging, particularly in immunocompromised patients, where CNS intervention might be unlikely to be performed. Despite the introduction of novel antifungals and surgical techniques, the overall prognosis and quality of life of these patients remain poor. High rates of mortality, morbidity (neurological sequelae) and scarcity of robust literature on CNS IMIs underscores the need for further research in this domain. There is a lack of comprehensive literature detailing the risk factors, clinical course, etiological agents and their resistance profile, treatment modalities, and disease outcome, making it challenging to draw definitive conclusions about the global epidemiology of CNS IMIs. The present study aimed to analyse the trend and epidemiology of CNS IMIs in adult and paediatric patients presenting to our tertiary care centre in Chandigarh, India.

## Methods

The present cohort study was conducted at a tertiary care public hospital in India. This was an ambispective study with a retrospective analysis of all patients of CNS IMIs from January 2004 to December 2015 (12 years), and a prospective analysis from January 2016 to March 2025 (9 years 3 months) resulting in a total study duration of 21 years 3 months.

All consecutive patients meeting the predefined inclusion criteria during the study period were eligible for enrolment irrespective of age and gender. Patients were included if they fulfilled the following criteria: Radiologically suspected intracranial infection or CNS space-occupying lesion suggestive of infection or abscess; and microbiological (direct smear and/or culture) and/or histopathological confirmation of invasive mould infection from CNS samples. Patients were identified from mycology and histopathology laboratory records, and relevant clinical (demographic, risk factors, clinical characteristics), imaging, management and outcome data were collected from the patient records. The prospective data collection was based on a case record form encompassing same clinical, microbiological, radiological, and therapeutic variables as those captured retrospectively, and follow up at 30 days after diagnosis for clinical and radiological assessment where feasible to evaluate treatment response and outcomes. Any missing information of follow up and outcome for the study was obtained telephonically from the patients. All eligible patients underwent brain biopsy and/or neurosurgical intervention (abscess drainage or excision or cerebrospinal fluid evaluation) as part of diagnostic confirmation and management. The participants received empirical/pre-emptive treatment as per the standard institutional protocol and targeted therapy after receiving the mycology reports. The empirical antifungal therapy is generally initiated with broad spectrum antifungal, amphotericin B until microbiological or histopathological confirmation is available. Subsequent modifications are made based on species identification, antifungal susceptibility, clinical response, and radiological findings. Amphotericin B is continued for those with confirmed mucormycosis while voriconazole is preferentially used for other mould infections. Combination therapy is reserved for refractory patients or severe disease, and treatment duration is individualised based on clinico-radiological response.

### Microbiological and histopathological analysis

The brain biopsy or abscess drainage samples received in the mycology laboratory were subjected to microscopic examination using 10% potassium hydroxide (KOH) calcofluor white mount. “Hyaline fungi” were defined as non-pigmented moulds with colourless or lightly stained hyphae on direct microscopy and histopathology, typically lacking melanin in the cell wall. “Melanized (dematiaceous) fungi” were defined as pigmented “dematiaceous” moulds characterised by brown to darkly pigmented hyphae or conidia on microscopy and histopathology due to the presence of melanin in the cell wall. For isolation of fungi, the samples were inoculated on Sabouraud dextrose agar (SDA) (incubated at 25 °C and 37 °C), blood agar (incubated at 37 °C), and brain heart infusion agar (BHIA) (incubated at 25 °C) and observed for mycelial growth for at least four weeks. Moulds were initially identified by phenotypic methods using the slide culture technique and lactophenol cotton blue (LCB) mount. The identity of the rare moulds was confirmed by amplifying the whole internal transcribed spacer region (ITS region) or large subunit [LSU (partial NL1/4)] of ribosomal DNA, followed by sequencing as per the previously published protocol.^11^ Additionally, a subset of biopsy samples underwent histopathological examination using periodic acid-Schiff (PAS) and hematoxylin and eosin (H&E) stains. Antifungal susceptibility testing (AFST) of filamentous fungi was performed in accordance with Clinical and Laboratory Standards Institute (CLSI) document M38.^12^

### Statistical analysis

Given the rarity of CNS invasive mould infections and the resulting low annual case counts, temporal trends were analysed using 6-year intervals to minimise year-to-year variability and facilitate clearer visualisation of long-term patterns. The descriptive data were analysed and presented as median (range), or frequencies/percentages as appropriate. Continuous variables with positively skewed distributions were summarized using median and interquartile range (IQR), with the IQR defined as the 25th–75th percentile. Chi-square and Fisher's exact test were employed for categorical variables and Student t-test was used for continuous variables. Odds ratios (ORs) with 95% confidence intervals (CIs) were calculated to estimate the strength of association between clinical, microbiological, and treatment-related variables and patient outcomes. Logistic regression analyses were performed to assess the association of mortality. Predictor variables were selected a priori based on domain knowledge and clinical relevance, with collinearity assessed and collinear variables excluded before model building. A two-tailed p-value ≤ 0.05 was considered as significant. Statistical analysis was performed using SPSS (version 25.0), and graphical representations were prepared using GraphPad Prism (version 9.0) and Excel (version 16.100.2).

### Ethics statement

Written informed consent was obtained from all prospectively enrolled patients or their legally authorised representatives, in accordance with institutional ethics committee approval. The study was approved by the Institute Ethics Committee under protocol number PGI/IEC/2011/697-98 dated 14-1-2012, Histo 15/IMEC/314 dated 1-4-2015, PGI/IEC/2018/000772 dated 21-5-2018, PGI/IEC/2018/001540 dated 8-10-2018.

### Role of funding source

The funders of the study had no role in study design, data collection, data analysis, data interpretation, or writing of the report.

## Results

There were a total of 1321 brain abscess/biopsy samples among which 127 patients (9.6%) were diagnosed to have moulds as aetiological agents of CNS infection. The number of patients demonstrated a progressive increase over time, with the highest case burden observed in the most recent study period [Supplementary Fig. S1].

The median age of the patients with CNS IMI was 30 years, with a predominance in males (71.7%). The median duration of symptoms was 15 days. Seventy four percent (n = 94) of patients were immunocompetent. Among the identifiable risk factors for immunological suppression (n = 33; 26.0%), the most common were steroid intake (n = 23; 18.1%), use of immunosuppressants (n = 17; 13.4%), malignancy (n = 15; 11.8%), renal transplant (n = 7; 5.5%) and chronic granulomatous disease (CGD) (n = 2; 1.6%). Head trauma (without open injury) was noted in two patients (1.6%) although it does not signify traumatic inoculation of fungus. Other comorbidities included uncontrolled diabetes mellitus (n = 14; 11.0% including six patients of mucormycosis), kidney disease (n = 10; 7.9%), liver disease (n = 5; 3.9%), tuberculosis (n = 1, 0.7%), and Tetralogy of Fallot (n = 1, 0.8%).

The detailed clinical features of CNS IMI in adults and paediatrics are provided in Table 1. The most common presentation was headache (61.4%), followed by seizures (50.4%), Most of the patients presented with a single space-occupying lesion (SOL) (66.1%). The radiological images of patients depicting single and multiple lesions are shown in Supplementary Fig. S2. The commonly afflicted site was the frontal lobe (61.4%) followed by the parietal lobe (40.9%).

**Table 1:** Comparison of clinico-mycological characteristics, treatment and outcome of adult and paediatric patients with CNS IMI.

Characteristic	Total (127) n (%)	Adult (100) n (%)	Paediatric (27) n (%)	Odds ratio (95% confidence interval)	p value
Age [Median (IQR)]	30 (27)	34 (23.25)	3 (1)	-	**--**
Females	36 (28.3)	25 (25.0)	11 (40.7)	–	Ref
Males	91 (71.7)	75 (75.0)	16 (59.3)	2.062 (0.846–5.028)	0.11
Symptoms					
Duration of symptoms (days) [median (IQR)]	15 (23)	15 (23)	12 (7)	–	**0.05**
Symptom duration <5 days	25 (19.7)	15 (15.0)	10 (37.0)	0.3 (0.115–0.779)	**0.01**
Symptom duration <30 days	97 (76.4)	75 (75.0)	22 (81.5)	0.682 (0.234–1.990)	0.484
Headache	78 (61.4)	71 (71.0)	7 (25.9)	0.143 (0.055–0.374)	**0.01**
Seizure	64 (50.4)	45 (45.0)	19 (70.4)	0.344 (0.138–0.860)	**0.02**
Fever	56 (44.1)	40 (40.0)	16 (59.3)	0.458 (0.193–1.089)	0.07
Altered sensorium	48 (37.8)	35 (35.0)	13 (48.1)	0.580 (0.246–1.370)	0.21
Vomiting	37 (29.1)	28 (28.0)	9 (33.3)	0.778 (0.313–1.935)	0.778
Hemiparesis	31 (24.4)	27 (27.0)	4 (14.8)	2.127 (0.673–6.716)	0.198
Focal neurological deficit	13 (10.2)	9 (9.0)	4 (14.8)	0.569 (0.161–2.012)	0.47
Risk factors					
No identifiable risk (immunocompetent)	94 (74.0)	79 (79.0)	15 (55.6)	3.010 (1.225–7.393)	**0.01**
Diabetes mellitus	14 (11.0)	13 (13.0)	1 (3.7)	3.885 (0.485–31.116)	0.20
Immunocompromised	33 (26.0)	21 (21.0)	12 (44.4)	-	**Ref**
Steroid intake	23 (18.1)	18 (18.0)	5 (18.5)	0.966 (0.323–2.893)	0.95
Immunosuppressants	17 (13.4)	10 (10.0)	7 (25.9)	0.317 (0.108–0.935)	**0.037**
Malignancy	15 (11.8)	7 (7.0)	8 (29.6)	0.179 (0.058–0.552)	**0.003**
Part of brain involvement					
Frontal lobe	78 (61.4)	57 (57.0)	21 (77.8)	0.379 (0.141–1.019)	**0.05**
Parietal lobe	52 (40.9)	37 (37.0)	15 (55.6)	0.433 (0.117–1.605)	0.21
Temporal lobe	24 (18.9)	19 (19.0)	5 (18.5)	1.032 (0.346–3.076)	0.95
Occipital lobe	13 (10.2)	8 (8.0)	5 (18.5)	0.383 (0.114–1.283)	0.12
Other sites	22 (17.3)	15 (15.0)	7 (25.9)	0.504 (0.182–1.399)	0.189
Number of lesions					
Single	84 (66.1)	72 (72.0)	12 (44.4)	–	Ref
Multiple	43 (33.9)	28 (28.0)	15 (55.6)	3.214 (1.339–7.716)	**0.009**
Medical treatment					
Medical therapy	116 (91.3)	92 (92.0)	24 (88.9)	1.437 (0.354–5.835)	0.61
Voriconazole	78 (61.4)	60 (60.0)	18 (66.7)	0.750 (0.307–1.835)	0.52
Amphotericin B	45 (35.4)	29 (29.0)	16 (59.3)	0.281 (0.116–0.678)	**0.005**
Amphotericin B deoxycholate	9 (7.1)	8 (8.0)	1 (3.7)	2.261 (0.270–18.910)	0.45
Liposomal amphotericin B	36 (28.3)	21 (21.0)	15 (55.6)	0.213 (0.087–0.522)	**0.001**
Surgical management					
Partial excision	56 (44.1)	38 (38.0)	18 (66.7)	–	Ref
Complete excision	71 (55.9)	62 (62.0)	9 (33.3)	0.306 (0.125–0.751)	**0.01**
Outcome					
Survived	88 (63.3)	71 (71.0)	17 (63.0)	–	Ref
Death	39 (30.7)	29 (29.0)	10 (37.0)	1.440 (0.590–3.516)	0.42

**Footnote:** Odds ratios (ORs) represent the odds of the specified characteristic in adult patients compared with paediatric patients, with paediatric patients used as the reference group. For the variable “number of lesions,” odds ratios represent paediatric patients compared with adult patients. Continuous variables are expressed as median (25th–75th percentile). Bold digits represent significant p value ≤ 0.05.

Rare cerebral sites exclusively involved were the ventricles (n = 5, 3.9%), cerebellum (n = 5; 3.9%), spinal vertebrae (n = 3; 2.4%), sagittal sinus (n = 2; 1.6%), caudate lobe (n = 2; 1.6), basal ganglia (n = 1; 0.8%), corpus callosum (n = 1; 0.8%), pituitary gland (n = 1; 0.8%), sphenoid sinus (n = 1; 0.8%), suprasellar region (n = 1; 0.8%) and thalamus (n = 1; 0.8%). No patients of systemic dissemination were observed.

Microscopy was positive in 126 patients (99.2%), with septate hyphae accounting for 89.0% (n = 113/126), and aseptate hyphae for 10.3% (n = 13/126) patients. The images of 10% KOH mount of hyaline and melanized (showing brown pigment and bulbous swellings) septate hyphae and aseptate hyphae are shown in Supplementary Fig. S3. Fungal culture yielded growth in 81.1% (n = 103) patients, *Aspergillus* species being the most common (n = 58/103, 56.3%) [*A. flavus* (n = 41/103; 39.8%), *A. fumigatus* (n = 13/103; 12.6%), *Aspergillus nidulans* (n = 3/103; 2.9%), *Aspergillus niger* (n = 1/103; 0.09%)] [Supplementary Fig. S4]. While *A. flavus* (n = 37; 44%) was the commonest in adults, *A. fumigatus* (n = 8; 42%) was predominant in children [Supplementary Table S1, Supplementary Fig. S9]. Overall, *C. bantiana* (n = 22/103; 21.36%) was the second most common etiologic agent after *A. flavus*. The thin very long melanized hyphae in microscopy usually indicates *C. bantiana,* with some hyphae showing small swollen areas as seen in Supplementary Fig. S3. Six-yearly trend of CNS IMI patients showed a noticeable decrease in infection by hyaline fungi and an increase in the infection by melanized fungi after 2021 (fractionally above the 2010–2015 levels) [Supplementary Fig. S1, Supplementary Fig. S10]. A rare melanized plant pathogen, *D. barringtoniae,* belonging to Family Didymosphaeriaceae, was reported as a human pathogen in a 65-year-old immunocompetent male who improved with voriconazole and partial excision of the temporal abscess. The 10% KOH mount showed dark septate hyphae with large bulbous swellings (soft pointer for phaeohyphomycosis) and culture on SDA showed creamish yellow colonies [Supplementary Fig. S5]. The lactophenol cotton blue mount showed melanized septate hyphae without sporulation [Supplementary Fig. S5]. The isolate was identified as *D. barringtoniae* by molecular method targeting both ITS and LSU regions of rDNA with 99.85% and 100% similarity respectively with *D. barringtoniae* MFLUCC 110422 Type Material (GenBank accession NR_111779, NG_042696). The sequences are submitted in GenBank database [accession under the accession no. PX285294 (ITS) and PX285293 (LSU)]. Species distribution in adults and paediatrics is given in Supplementary Tables S1 and S2. The AFST data of representative isolates are given in Table 2. AFST demonstrated variable minimum inhibitory concentration (MIC) ranges across mould species, with lower azole MICs observed for most *Aspergillus* spp. and melanized fungi.

**Table 2:** Distribution (range) of minimum inhibitory concentrations of amphotericin B and triazoles and minimum effective concentrations of echinocandins (in μg/ml) against fungi isolated from central nervous system invasive mould infection patientspatients during the study (2004–2025).

Isolate name	AMB	VRC	ITR	POS	CAS	ANI	MICA
*C. bantiana* (n = 7)	0.12–8	0.03–0.12	0.03–4	0.03–1	0.03–16	0.03–16	0.12–16
*C. hawaiiensis* (n = 1)	0.12	0.12	–	0.25	0.25	0.25	–
*C. lunata* (n = 1)	0.12	0.12	–	0.12	0.06	0.06	–
*A. fusispora* (n = 3)	0.5–4	0.03–0.12	0.12–0.25	0.12–0.5	8–16	8–16	2–16
*S. apiospermum* (n = 1)	2	0.25	1	1	–	–	–
*A. flavus* (n = 4)	4–8	0.25–1	0.5–2	0.25–2	0.03–0.5	0.03–8	0.03
*A. fumigatus* (n = 4)	1–4	0.25–1	0.25–1	0.25–0.5	0.03–0.25	0.03–0.25	0.03
*A. nidulans* (n = 1)	4	4	0.25	0.25	0.25	0.25	0.03
*S. commune* (n = 1)	4	0.5	0.12	0.25	0.03	0.03	0.03
*F. oxysporum* (n = 1)	2	8	16	8	16	4	16
*C. sphaerospermum* (n = 1)	1	1	2	0.25	1	8	8
*R. arrhizus* (n = 1)	2	–	8	2	–	–	–
*R. microsporus* (n = 1)	0.5	–	4	4	–	–	–

CLSI breakpoints are not available for these isolates. Interpretation of MIC values of *A. flavus* and *A. fumigatus* done according to epidemiological cut off values (ECV) (CLSI) according to which *A. fumigatus* (one isolate each) showed higher MIC (than ECV) against amphotericin B and voriconazole while one *A. flavus* isolate showed elevated MIC (than ECV) against amphotericin B, itraconazole, posaconazole and caspofungin; and one isolate showed elevated MIC (than ECV) against amphotericin B alone.

Abbreviations: ANI: Anidulafungin, AMB: Amphotericin B, CAS: Caspofungin, ITR: Itraconazole, MICA: Micafungin, POS: Posaconazole, VRC: Voriconazole.

Histopathological evaluation was available for 56 patients, which included morphologically documented hyaline septate hyphae (n = 41), aseptate hyphae (n = 8) and melanized (dematiaceous or phaeoid or pigmented) hyphae (n = 7). Hyaline septate hyphae patients demonstrated granulomatous reaction [(n = 27; *A. flavus* (n = 19), *A. fumigatus* (n = 3), *A. niger* (n = 1), culture negative (n = 4)] and acute necrotizing inflammatory response with the formation of microabscesses [(n = 14; *A. flavus* (n = 4), *A. fumigatus* (n = 3), *Fusarium oxysporum* (n = 1), *Purpureocillium lilacinum* (n = 1), culture negative (n = 5)] [Supplementary Fig. S6]. While a typical granulomatous response consisted of a collection of epithelioid cells admixed with giant cells with negative shadows of fungal hyphae, in an equal proportion of patients, these giant cells were instead loosely scattered, forming ill-formed granulomas. Periodic-acid Schiff's (PAS) stain highlighted the slender, septate hyphae within the giant cells and within the surrounding fibrotic tissue. Adjacent brain parenchyma in most of the patients displayed coarse reactive gliosis and fibrosis with chronic lymphomononuclear inflammation. In patients particularly encountered as dural/skull-based lesions, dense fibrotic/sclerotic response was evident in the background of the granulomatous reaction, which notably demonstrates a chronic nature of *Aspergillus* infection. The patients localized deep within the brain parenchyma were dominated by a necrotizing inflammatory response with surrounding necrosis and the presence of invasive fungal hyphae.

Morphologically, mucormycosis (n = 8) was characterized by the presence of bland necrosis devoid of an inflammatory response within which numerous broad, aseptate, obtuse-angled hyphae were observed associated with vasculitis and angioinvasion. Features of multiple loose epithelioid granulomas with many giant cells were also noticed in few patients. The fungal hyphae could be easily highlighted with Grocott's methenamine silver stain.

Patients with melanized fungi (n = 7) (*C. bantiana*, n = 6; *Chaetomium*, n = 1) featured an admixture of a granulomatous reaction and formation of microabscesses. In a single case, formation of multiple cysts bordered by a fibrotic wall with palisading histiocytes, and multi-nucleated giant cells was also evident [Supplementary Fig. S7]. Notably, angioinvasion or vasculitis was not regular feature with these fungi. All the patients of phaeohyphomycosis demonstrated melanin producing pigmented fungi accentuated on Masson Fontana stain. These fungi showed thin branching hyphae with prominent bulbous swellings and septate constrictions.

Voriconazole (61.4%) was the most commonly used antifungal followed by amphotericin B (35.4%). Combined use of liposomal amphotericin B and voriconazole was noted in 16.37% (n = 19) patients. Surgical intervention was performed in the form of complete excision of the lesion (55.9%) or partial excision (44.1%).

The overall mortality was observed in 30.7% (n = 39) which showed a decreasing trend over the years [Supplementary Fig. S1, Supplementary Fig. S11]. The species wise mortality in adult and paediatric patients is given in Supplementary Fig. S9. Headache and vomiting were significantly more frequent among survivors than non-survivors [Table 3]. Complete excision of the lesion was significantly more frequent among survivors than non-survivors [Table 3]. The logistic regression analysis identified the lack of headache and partial excision of the lesion as independent risk factors for poor clinical outcomes [Table 4]. Kaplan–Meier survival curve analysis showed a better outcome in patients undergoing complete excision of the lesion (p = 0.02) and a worse outcome in patients with lesions in the parietal lobe (p = 0.048) [Supplementary Fig. S8].

**Table 3:** Comparison of clinical characteristics, treatment and outcome of survivors and non-survivors with central nervous system invasive mould infection.

Characteristic	Total (127)	Non-survivors (39)	Survivors (88)	Odds ratio (95% confidence interval)	p value
Age [Median (IQR)]	30 (27)	31 (32)	30 (25.25)	–	0.32
Adult	100 (78.7)	29 (74.4)	71 (80.7)	–	Ref
Paediatric	27 (21.3)	10 (25.6)	17 (19.3)	1.440 (0.590–3.516)	0.42
Females	36 (28.3)	11 (28.2)	25 (28.4)	–	Ref
Males	91 (71.7)	28 (71.8)	63 (71.6)	0.990 (0.429–2.287)	0.98
Symptoms					
Duration of symptoms (days) (mean ± SD)	15 (23)	15 (17.5)	15.5 (25.5)	–	0.08
Symptom duration <5 days	25 (19.7)	10 (25.6)	15 (17.0)	0.596 (0.240–1.478)	0.26
Symptom duration <30 days	97 (76.4)	32 (82.1)	65 (73.9)	0.618 (0.240–1.592)	0.31
Headache	78 (61.4)	17 (43.6)	61 (69.3)	2.924 (1.342–6.368)	**0.007**
Seizure	64 (50.4)	22 (56.4)	42 (47.7)	0.706 (0.330–1.507)	0.36
Fever	56 (44.1)	19 (48.7)	37 (42.0)	0.764 (0.358–1.628)	0.48
Altered sensorium	48 (37.8)	18 (46.2)	30 (34.1)	0.603 (0.280–1.301)	0.19
Vomiting	37 (29.1)	7 (17.9)	30 (34.1)	2.365 (0.934–5.987)	0.06
Hemiparesis	31 (24.4)	9 (23.1)	22 (25.0)	1.111 (0.457–2.699)	0.81
Focal neurological deficit	13 (10.2)	5 (12.8)	8 (9.1)	0.680 (0.207–2.229)	0.52
Risk factors					
No identifiable risk (immunocompetent)	94 (74.0)	28 (71.8)	66 (75.0)	1.179 (0.505–2.752)	0.70
Diabetes mellitus	14 (11.0)	4 (10.3)	10 (11.4)	1.122 (0.329–3.823)	0.85
Kidney disease (CKD)	10 (7.9)	4 (10.3)	6 (6.8)	0.640 (0.170–2.410)	0.51
Liver disease	5 (3.9)	1 (2.6)	4 (4.5)	1.810 (0.196–16.737)	0.60
Immunocompromised	33 (26.0)	11 (28.2)	22 (25.0)	–	Ref
Steroid intake	23 (18.1)	10 (25.6)	13 (14.8)	0.503 (0.198–1.273)	0.14
Immunosuppressants	17 (13.4)	6 (15.4)	11 (12.5)	0.786 (0.268–2.302)	0.66
Malignancy	15 (11.8)	5 (12.8)	10 (11.4)	0.872 (0.277–2.744)	0.81
Renal transplant	7 (5.5)	3 (7.7)	4 (4.5)	0.571 (0.122–2.684)	0.47
Primary immunodeficiency disease (Chronic granulomatous disease)	2 (1.6)	1 (2.6)	1 (1.1)	0.437 (0.027–7.168)	0.56
Part of brain involvement					
Frontal lobe	78 (61.4)	23 (59.0)	55 (62.5)	1.317 (0.577–3.004)	0.51
Parietal lobe	52 (40.9)	20 (51.3)	32 (36.4)	0.331 (0.095–1.160)	0.08
Temporal lobe	24 (18.9)	5 (12.8)	19 (21.6)	1.872 (0.644–5.444)	0.24
Occipital lobe	13 (10.2)	6 (15.4)	7 (8.0)	0.475 (0.149–1.521)	0.21
Other sites	22 (17.3)	5 (12.8)	17 (19.3)	1.628 (0.554–4.783)	0.37
Number of lesions					
Single	84 (66.1)	22 (56.4)	62 (70.5)	–	Ref
Multiple	43 (33.9)	17 (43.6)	26 (29.5)	1.843 (0.844–4.025)	0.12
Medical treatment					
Medical therapy	116 (91.3)	35 (89.7)	81 (92.0)	1.322 (0.364–4.808)	0.67
Amphotericin B	45 (35.4)	15 (38.5)	30 (34.1)	0.828 (0.379–1.808)	0.63
Amphotericin B deoxycholate	9 (7.1)	3 (7.7)	6 (6.8)	0.878 (0.208–3.707)	0.86
Liposomal amphotericin B	36 (28.3)	12 (30.8)	24 (27.3)	0.844 (0.369–1.928)	0.68
Voriconazole	78 (61.4)	23 (59.0)	55 (62.4)	1.159 (0.537–2.505)	0.70
Surgical management					
Partial excision	56 (44.1)	27 (69.2)	29 (33.0)	–	Ref
Complete excision	71 (55.9)	12 (30.8)	59 (67.0)	0.218 (0.097–0.492)	**0.001**

**Footnote:** Odds ratios (ORs) represent the odds of death (non-survival) for each variable. Survivors were used as the reference group unless otherwise specified. Continuous variables are expressed as median (25th–75th percentile). Bold digits represent significant p value ≤ 0.05.

**Table 4:** Logistic regression analysis of risk factors for unfavourable clinical outcomes in patients with central nervous system invasive mould infection.

Variable (frequency count)	Odd's ratio	95% CI	p value
		Lower-upper	
Headache (n = 78)	0.429	0.188–0.979	**0.044**
Partial excision (n = 56)	3.922	1.703–9.034	**0.001**
Single lesion (n = 84)	0.622	0.267–1.447	0.270

**Footnote:** Multivariable logistic regression analysis showing factors independently associated with unfavourable clinical outcome. Odds ratios (ORs) represent the odds of an unfavourable outcome. Bold digits represent significant p value ≤ 0.05.

Overall comparison of clinical and mycological features of CNS IMI among adult and paediatric patients is shown in Table 1. Children were significantly more likely to have underlying immunocompromising conditions [Table 1]. Malignancy was strongly associated with paediatric disease [Hodgkin's lymphoma (1); AML (7)] (29.6% vs 7.0%), reflected by substantially lower odds among adults [Acute lymphoblastic leukaemia (ALL), (1); acute myeloid leukaemia (AML) (1), meningioma (1), glioblastoma (1), Hodgkin's Lymphoma (1), pituitary adenoma (2)]. Headache was markedly less frequent in paediatric patients, whereas seizures were more. Radiologically, paediatric patients were more likely to have multiple lesions, whereas adults had over three times higher odds of a single lesion [Table 1]. Species distribution according to risk factors in both adults and children is given in Supplementary Table S1. All three patients of *A. nidulans* were reported in children, of which two had immune dysfunction [T-cell ALL (n = 1) and CGD (n = 1)] [Supplementary Table S1]. Partial excision and the use of amphotericin B including liposomal amphotericin B were more common in paediatric patients.

Overall comparison of clinical and demographic characteristics of CNS IMI due to melanized and hyaline fungi (aseptate excluded) is shown in Table 5. Patients with melanized fungal infection were significantly older than those with hyaline fungi and paediatric patients were markedly less likely to have melanized fungi compared with hyaline fungi. Underlying kidney disease, renal transplantation and steroid exposure were strongly associated with melanized fungal infection [Table 5]. Clinical presentation differed significantly between groups. Headache, fever, altered sensorium, and hemiparesis were significantly more frequent in patients with melanized fungi. The radiological imaging features of cerebral phaeohyphomycosis (*C. bantiana* and *Chaetomium* spp.) and hyalohyphomycosis (*A. fumigatus*) are shown in Supplemenatry Fig. S2. Surgical management differed significantly: complete excision was less frequently performed in melanized fungal infection and was associated with lower odds of melanized infection compared with partial excision.

**Table 5:** Comparison of clinical characteristics, treatment and outcome of patients with central nervous system invasive mould infection due to melanized (cerebral phaeohyphomycosis) vs hyaline fungi (cerebral hyalohyphomycosis).

Characteristic	Total (Aseptate excluded) (114)	Melanized fungi (36) (cerebral phaeohyphomycosis)	Hyaline fungi (78) (cerebral hyalohyphomycosis) (excluding mucormycosis)	Odds ratio (95% Confidence interval)	p value
Age [Median (IQR)]	30 (24)	33.5 (25)	27 (25)	–	**0.03**
Adult	89 (78.1)	34 (94.4)	55 (70.5)	–	Ref
Paediatric	25 (21.9)	2 (5.6)	23 (29.5)	0.141 (0.031–0.635)	**0.01**
Females	33 (28.9)	6 (16.7)	27 (34.6)	–	Ref
Males	81 (71.7)	30 (83.3)	51 (65.4)	0.378 (0.140–1.020)	**0.05**
Symptoms					
Duration of symptoms (days) [Median (IQR)]	15 (20)	10 (11.5)	15 (23)	–	0.19
Symptom duration <5 days	24 (21.1)	7 (19.4)	17 (21.8)	1.155 (0.431–3.092)	0.77
Symptom duration <30 days	88 (77.2)	28 (77.8)	60 (76.9)	0.952 (0.370–2.453)	0.919
Headache	70 (61.4)	28 (77.8)	42 (53.8)	0.333 (0.135–0.822)	**0.01**
Seizure	61 (53.5)	20 (55.6)	41 (52.6)	0.886 (0.401–1.96)	0.76
Fever	51 (44.7)	23 (63.9)	28 (35.9)	0.317 (0.139–0.720)	**0.006**
Altered sensorium	45 (39.5)	20 (55.6)	25 (32.1)	0.377 (0.168–0.849)	**0.01**
Vomiting	36 (31.6)	13 (36.1)	23 (29.5)	0.740 (0.321–1.707)	0.48
Hemiparesis	28 (24.6)	14 (38.9)	14 (17.9)	0.344 (0.142–0.833)	**0.01**
Focal neurological deficit	13 (11.4)	4 (11.1)	9 (11.5)	1.043 (0.299–3.643)	0.94
Risk factors					
No identifiable risk (immunocompetent)	83 (72.8)	24 (66.7)	59 (75.6)	1.553 (0.654–3.686)	0.31
Kidney disease	10 (8.8)	9 (25.0)	1 (1.3)	0.039 (0.005–0.332)	**0.003**
Diabetes mellitus	8 (7.0)	4 (11.1)	4 (5.1)	0.432 (0.102–1.837)	0.25
Liver disease	5 (4.4)	3 (8.3)	2 (2.6)	0.289 (0.046–1.814)	0.18
Immunocompromised	31 (27.2)	12 (33.3)	19 (24.4)	–	Ref
Steroid intake	21 (18.4)	11 (30.6)	10 (12.8)	0.334 (0.127–0.883)	**0.02**
Immunosuppressants	16 (14.0)	6 (16.7)	10 (12.8)	0.735 (0.245–2.208)	0.58
Malignancy	14 (12.3)	2 (5.6)	12 (15.4)	3.091 (0.654–14.608)	0.15
Renal transplant	7 (6.1)	6 (16.7)	1 (1.3)	0.065 (0.007–0.562)	**0.01**
Part of brain involvement					
Frontal lobe	70 (61.4)	18 (50.0)	52 (66.7)	1.500 (0.616–3.650)	0.37
Parietal lobe	49 (43.0)	20 (55.6)	29 (37.2)	0.432 (0.102–1.837)	0.25
Temporal lobe	21 (18.4)	7 (19.4)	14 (17.9)	0.906 (0.331–2.483)	0.84
Occipital lobe	11 (9.6)	4 (11.1)	7 (9.0)	0.789 (0.216–2.887)	0.72
Other sites	21 (18.4)	3 (8.3)	18 (22.1)	3.300 (0.905–12.036)	0.07
Number of lesions					
Single	75 (65.8)	24 (66.7)	51 (65.4)	–	Ref
Multiple	39 (34.2)	12 (33.3)	27 (34.6)	0.944 (0.410–2.177)	0.89
Medical treatment					
Medical therapy	104 (91.2)	33 (91.7)	71 (91.0)	0.922 (0.224–3.792)	0.91
Voriconazole	74 (64.9)	23 (65.7)	51 (64.6)	0.893 (0.388–2.054)	0.79
Amphotericin B	34 (29.8)	13 (36.1)	21 (26.9)	0.652 (0.280–1.516)	0.32
Amphotericin B deoxycholate	9 (7.9)	4 (11.1)	5 (6.4)	0.548 (0.138–2.176)	0.39
Liposomal amphotericin B	25 (21.9)	9 (25.0)	16 (20.5)	0.774 (0.304–1.969)	0.59
Surgical management					
Partial excision	49 (43.0)	22 (61.1)	27 (34.6)	–	Ref
Complete excision	65 (57.0)	14 (38.9)	51 (65.4)	0.337 (0.149–0.762)	**0.009**
Outcome					
Survived	80 (70.2)	23 (63.9)	57 (73.1)	–	Ref
Death	34 (29.8)	13 (36.1)	21 (26.9)	1.534 (0.659–3.569)	0.32

**Footnote:** Odds ratios (ORs) represent the odds of the specified characteristic in patients with melanized fungal infection (cerebral phaeohyphomycosis) compared with hyaline fungal infection (cerebral hyalohyphomycosis), with hyaline fungal infection used as the reference group. Continuous variables are expressed as median (25th–75th percentile). Bold digits represents significant p value ≤ 0.05.

Further, we also compared the characteristics of CNS IMI caused by the most common hyaline (*Aspergillus* spp.) and melanized fungi (*C. bantiana*) [Supplementary Table S3]. All patients of *C. bantiana* were exclusively present in adult population. Among the clinical features, headache and fever were significantly more frequent in *C. bantiana* infection than in *Aspergillus* infection. Steroid intake, renal transplant and kidney disease were significantly associated with *C. bantiana* infection.

A total of 13 patients of CNS mucormycosis were enrolled (adults, n = 11; paediatrics, n = 2) [Supplementary Table S2]. All showed aseptate hyphae in microscopy while culture was positive in 53.8% (n = 7) patients, *Rhizopus* *arrhizus* (n = 5) and *Rhizopus* *microsporus* (n = 2) [Supplementary Fig. S4, Supplementary Table S1]. DM (n = 6) was the commonest risk factor (adults, n = 5 and paediatrics, n = 1). A 4-year-old female was diagnosed with ALL and a 46-year-old female had HLAB27 + arthritis and was on steroids and sulfasalazine. One patient with DM had a previous history of rhino-orbital-cerebral mucormycosis (ROCM). Five patients did not exhibit any identifiable risk factor for mucormycosis. The radiological imaging of cerebral mucormycosis is shown in Supplementary Fig. S2.

## Discussion

CNS IMI represents a serious and life-threatening manifestation of invasive fungal infections, particularly in regions like India, where the tropical climate makes it conducive to these infections. Despite its significance, data on CNS IMI remains limited, largely confined to scattered case reports or small case series. Our study bridges this gap by providing comprehensive data on CNS IMI patients over two decades from a tertiary care hospital in India. The observed incidence of CNS IMI in our study was 9.6%, a notably higher figure than other studies from India, which reported an incidence ranging from 0.99 to 1.7% over 24–38 years.^7^^,^^13^ The data from both the centres were derived from hospital-based retrospective analyses of fungal brain abscesses managed by neurosurgical services at a large tertiary-care referral institute, similar to our institute. Furthermore, improved diagnostic facilities, increased neurosurgical interventions, better sampling, and enhanced referral patterns to the tertiary-care centre can lead to higher incidence at our centre. Additionally, the geographical variation in incidence could be attributed to differences in environmental factors, occupational exposure, availability of mycology laboratories or under-reporting of the aetiology of infection. Climate change may affect the adaptation of the fungi to human body temperature leading to increase in number of patients particularly those by melanized fungi which exhibit thermotolerance.^14^ It is interesting to note that though total number of patients and infection by melanized fungi have increased over the years, the mortality has decreased which might be due to increased awareness, early diagnosis, availability of molecular methods and prompt initiation of appropriate antifungal therapy. Continuous surveillance is essential to understand the epidemiology and changing trends of CNS IMI over time.

We observed a higher prevalence of CNS IMI in young adult males, possibly due to increased outdoor exposure, given that fungi are ubiquitous in the environment, with geographical variation in spore burden.^15^ Interestingly, most of the patients in our study (74%) did not have any identifiable risk factor (immunocompetent), which raises questions about host genetic susceptibility and the potential impact of global warming and climate change on fungal virulence.^16^ Among the risk factors, steroid intake, malignancy, use of immunosuppressants, renal transplantation and CGD were notable as they cause immune dysfunction and increase host susceptibility to fungal infections. The susceptibility of the transplant recipients to infection by melanized fungi (*C. bantiana* and *Acrophialophora* *fusispora*) is interesting to note and needs further investigation. Significant association of kidney disease, renal transplant and steroids with *C. bantiana* infection warrants exploring virulence of this fungus in these hosts. Malignancy particularly ALL and AML, was a significant risk factor in children, which is also described in studies from Europe, where these children are more prone to develop CNS invasive fungal diseases.^17^ DM, a well-known risk factor for ROCM, was noticed in 6/13 patients of CNS mucormycosis.^18^

The mode of entry of fungal pathogens into the CNS is not fully understood. The anatomical location of a brain abscess is partly influenced by the route of infection transmission; for instance, paranasal sinusitis is often associated with frontal lobe abscesses, while otitis media and mastoiditis are associated with temporal lobe or cerebellar abscesses. In rare patients, direct extension through the eye or middle ear may occur. We observed the frontal lobe as the most commonly afflicted site, followed by the parietal, temporal, and occipital lobes. The agent likely gains entry via inhalation of fungal spores, with subsequent hematogenous dissemination to the CNS.^19^ These findings concur with previous findings from India, where frontal lobe involvement was noted in 48% of the patients while parietal and occipital regions were less affected.^13^ Solitary brain abscesses typically result from the local spread of infection, from adjacent structures such as the paranasal sinuses, the middle ear or from an infected traumatic or surgical wound. Hematogenous spread from a distant focus such as the heart or lung, forms the clinical setting for multiple abscesses and these lesions usually occur in the frontal or temporal lobes. In the vast majority of patients with cerebral aspergillosis, the CNS is invaded by hematogenous spread from primary sites of infection (e.g., lungs or sinuses) or by destructive extension of invasive fungal sinusitis.^20^ Interestingly, multiple lobe involvement was noticed in four patients of CNS mucormycosis (4/13). Notably, a single case had a previous history of rhino-orbital mucormycosis.

In our cohort, the predominant clinical features included headache (61.4%), seizures (50.4%), fever (44.1%), altered sensorium (37.8%), vomiting (29.1%) and hemiparesis (n = 24.4%). The lower incidence of headache in children reflects their limited ability to effectively communicate symptoms. Notably, frontal lobe involvement, more commonly involved in present study also, often manifests with headache, drowsiness, and deterioration of mental status or aphasia, together with hemiparesis and unilateral motor signs.^19^ Clinical manifestations varied based on abscess location, size, pathogen virulence, and host immune response, though symptoms were frequently nonspecific, contributing to diagnostic delays (median: 15 days in our study). The duration of symptoms was also shorter in children manifesting as a high rate of seizures and fever as compared to adults. Headache and vomiting were significantly more common among survivors, possibly due to early clinical presentation and diagnosis, allowing prompt management. Additionally, fever, headache, altered sensorium, and hemiparesis, were more frequently observed in infections caused by melanized fungi compared to hyaline fungi, which might possibly be related to the virulence due to melanin production.^21^ CNS mucormycosis also depicted higher frequency of fever and headache.

Among the etiologic agents, *Aspergillus* spp., especially *A. flavus* (39.8%), was the most common species isolated, followed by *C. bantiana* (21.36%), a highly neurotropic fungus. This contrasts with South Indian data, where *C. bantiana* (44.8%) was the predominant isolate, followed by *Aspergillus* spp. (20.6%).^7^ In another study from a tertiary care centre in India, mucormycosis was observed as the most common mould infection followed by aspergillosis.^6^ Baddley et al. from the USA have reported *A. fumigatus* as the most common species among brain abscess specimens in transplant recipients, while Torre-Cisneros et al. noted *A. flavus* comprising 24% of CNS aspergillosis patients among organ transplant patients in the Western USA.^22^^,^^23^ The differences in species could be attributed to variations in fungal prevalence within different geographical niches. Worldwide, *A. fumigatus* is the most prevalent species in the environment followed by *A. flavus* and *Aspergillus terreus*. Both *A. flavus* and *A. fumigatus* have been previously reported to be abundant in Indian environment.^24^ Infection by *A. nidulans* exclusively in children is noteworthy emphasizing the investigation of host factors and virulence factors of this species. CGD is a well-known risk factor for developing *A, nidulans* infection.^25^ Of three paediatric patients infected with *A. nidulans*, risk factors included CGD (n = 1) and ALL (n = 1); while infection in a patient with no identifiable risk factor needs to be evaluated for any immunodeficiency disease. *C. bantiana* is reported in significant number of patients from Asian countries, particularly India, possibly due to its thermotolerant nature enabling its survival in tropical regions or host genetic susceptibility.^5^ Interestingly, *C. bantiana* infection was exclusively present in adults, possibly due to their higher environmental exposure to the fungus which is believed to inhabit plant material and soil. However, further exploration of its environmental niche is warranted. The early diagnosis of the disease remains a significant challenge due to its rarity and the presence of overlapping symptoms and signs of the disease. We report *D. barringtoniae* as a cause of human infection for one of the first time in literature.^26^ The genus *Deniquelata* consists of five species: *D. barringtoniae* (type species), *D. hypolithi*, *D. quercina*, *D. vittalii* and *D. yunnanensis. D. barringtoniae* is reported to be a plant pathogen causing leaf spots in *Barringtonia asiatica.*^26^ Its infection in an immunocompetent patient indicates either its higher virulence or unexplored host genetic susceptibility factors. Among Mucorales, *R. arrhizus* was the commonest agent as described previously.^27^

Histopathological examination of the fungal brain abscess is crucial, as the histopathological changes in the brain vary with the disease progression. In the initial 1–2 weeks, lesions are poorly demarcated, exhibiting localized cerebral edema and acute inflammatory changes. By 2–3 weeks, the abscess undergoes encapsulation, with necrosis and liquefaction evident.^28^ We observed features of granulomas predominantly in invasive aspergillosis, while microabscesses were found in invasive aspergillosis and IMI by other hyaline moulds like *P. lilacinum, F. oxysporum* and melanized fungi. Necrosis and angioinvasion were features of mucormycosis. In a case series on cerebral phaeohyphomycosis, diffuse micro abscesses were consistently observed, which were similarly noted in our patients.^29^

Majority of the CNS IMI patients presented as single lesions (66.1%) in both paediatric (44.4%) and adult (72.0%) patients irrespective of infections by melanized or hyaline fungi. However majority of CNS mucormycosis patients exhibited single lesions (9/13). Radiological characteristics of fungal infections are a direct extrapolation of their pathological aspects. Fungal granulomas are typically peripheral enhancing lesions with a variable degree of central necrosis. The capsule often shows T2 hypointense signal with thick, irregular nodular enhancement. Susceptibility changes and diffusion restriction are often seen along the rim. Frank abscesses may show central diffusion restricting contents. Due to associated vasculitis in the small vessels of the brain parenchyma harbouring the fungal abscess, microhaemorrhages may often be seen, which are indicative of their angioinvasive character. Also, these lesions show significant perilesional oedema.

Management of fungal brain abscess typically involves a combination of surgical excision and antifungal therapy, as recommended by European Society of Clinical Microbiology and Infectious Diseases (ESCMID) and European Confederation of Medical Mycology (ECMM) joint guidelines and Infectious Diseases Society of America (IDSA).^30^^,^^31^ In our cohort, complete excision was performed in 55.9% of patients, though partial excision was significantly more common in children, possibly due to the surgical challenges of paediatric neurosurgery. The identification of the fungus is essential for prescribing antifungal therapy. AFST of individual fungi guides the management particularly melanized fungi having variable antifungal susceptibility pattern and lack of breakpoints for interpretation; and emerging drug resistance in *Aspergillus* spp. IDSA recommends voriconazole or liposomal amphotericin B for the treatment of CNS aspergillosis. Amphotericin B is the drug of choice for treating mucormycosis and *S. commune* infection.^31^ First-line treatment for infection by *Scedosporium* spp., *Fusarium* spp., and melanized fungi is voriconazole due to lower MICs and better CNS penetration.^31^^,^^32^ Alternative treatment includes a combination of voriconazole with other antifungals. The higher use of liposomal amphotericin B in children may reflect concerns over the side effects of other antifungals or the inability to use voriconazole below 2 years of age, in paediatric patients. Liposomal amphotericin B is well tolerated in paediatric patients.^17^

The overall mortality in our study was 30.7% in contrast to another study from South India, which reported 62% fatality.^7^ Mortality was comparatively higher in paediatric patients (37%) than adults (29%) though not statistically significant, which could be attributed to the difficulty in performing surgical interventions and a higher rate of partial excision in children leading to poor prognosis. Other previously reported factors contributing to mortality particularly in paediatric haematology oncology patients were underlying disease, chemotherapy phase and host factors.^17^ We observed the absence of headache and partial excision of the lesion as independent risk factors for unfavourable clinical outcomes among patients with CNS IMI, potentially due to delay in symptoms eventually causing delay in presentation to the hospital and challenges in surgical management. Kruthika et al., also reported high mortality (55.5%) among patients with aspiration without excision than patients undergoing craniotomy with excision/decompression (50%).^7^ Chakrabarti et al. observed that complete excision of the lesion was associated with better outcomes, particularly in immunocompetent patients infected with *C. bantiana*.^5^ Though not statistically significant, the higher mortality in infections by melanized fungi (36.1%) than those by hyaline fungi (26.9%) needs to be investigated for the attributable mortality in these patients. CNS mucormycosis exhibited 38.5% mortality as reported in literature.^27^

Our study was ambispective in nature, and there were limitations due to a lack of data on serological biomarkers, and the unavailability of antifungal susceptibility testing for some fungal isolates. Although this study represents one of the larger single-centre cohorts of CNS IMIs, the ambispective design and small sample sizes within certain subgroups limit the strength of inferential analyses. Integrating retrospective with prospective data sources can create selection and temporal biases, data availability disparities and inconsistencies (often greater for retrospective than with prospective data) and the temporal difference between the two sources can create confounding problems. Nevertheless, the data provide an important contemporary overview of the clinical and mycological spectrum of CNS IMI and serve as a valuable reference framework for clinicians, both infectious disease physicians and paediatricians, as well as a foundation for future multicentre and prospective studies.”

In conclusion, CNS IMI, although an uncommon disease remains a life-threatening condition, particularly among immunocompromised patients, necessitating early clinical suspicion and prompt diagnostic efforts to improve outcomes. This study underscores the increasing significance of CNS IMI, particularly in lower-income and middle income countries with tropical climates with emergence of rare pathogens (like *D. barringtoniae*) and affecting immunocompetent hosts as well. The predominance of cerebral phaeohyphomycosis in adults, particularly renal transplant recipients, and cerebral hyalohyphomycosis in children with haematological malignancy warrants further exploration of host-pathogen dynamics. The heightened mortality associated with CNS IMIs emphasises the critical need for early suspicion, accurate diagnosis, and targeted antifungal therapy. Combined surgical excision and antifungal treatment remain integral to improving patient outcomes.

## Contributors

HK, SRM, RK and HA did the literature search; HK, SRM and AC did the study design; HK, SRM, RK, HA, PS, DB, KG, DC, CKA, ATK, MM, SM, RC, MK, HS, AS, AA, SV, SKG, SJ, AT, SVy, VP, AG, AC did the data collection; HK, RK, HA, KG, DC, CKA, SV did the data analysis; HK, SRM, RK, HA, PS, DB, KG, DC, CKA, ATK, MM, SM, RC, MK, HS, AS, AA, SV, SKG, SJ, AT, SVy, VP, AG, AC did the project management; HK, SRM, RK, HA, KG, DC, CKA, SVy, AC did the data interpretation; HK, RK, HA, KG, DC, CKA, SV accessed and verified the data; HK, SRM and RK wrote the first draft of the manuscript. All authors had access to the data and critically reviewed the manuscript for important intellectual content and approved the final version. HK SRM, and RK had the final responsibility to submit for publication.

## Data sharing statement

Data is available on request from the corresponding author.

## Declaration of interests

We declare no competing interests.
