# Supplementary material for: Invasive mould infections of the central nervous system in the Indian population: a cohort study (2004–2025)
Source: Lancet Reg Health Southeast Asia. 2026 Mar 4;46:100736. doi: 10.1016/j.lansea.2026.100736 (PMC12969336; doi:10.1016/j.lansea.2026.100736)
Supplement: Supplementary Tables and Figures [file mmc1.docx]

**Appendix A Supplementary data (1)**

**Supplementary tables (S1-S3) and figures (S1-S11)**

**Supplementary Table S1.** Risk factor wise detail of fungi in patients with central nervous system invasive mould infection (n=113) (excluding mucormycosis).*

| Risk factor (n) | No. of culture positive cases | Adults | Paediatrics |
| --- | --- | --- | --- |
| No identifiable risk (immunocompetent) (n=83) | 70 | *A. flavus* (33), *C. bantiana* (15), *A. fumigatus* (3), *S. apiospermum* (2), *A. fusispora* (1), *Chaetomium spp.* (1), *C. sphaerospermum* (1), *C. lunata* (1), *D. barringtoniae* (1), *F. solani* (1), *S. commune* (1) | *A. fumigatus* (4), *A. flavus* (2), *A. nidulans* (1), *E. werneckii* (1), *F. oxysporum* (1), *P. lilacinus* (1) |
| Kidney disease (n=10) |  | *C. bantiana* (6), *A. fumigatus* (1), *A. fusispora* (1), *S. apiospermum* (2) | *-* |
| Diabetes mellitus (n=8) | 6 | *C. bantiana* (3), *A. flavus* (2), *A. fumigatus* (1) | *-* |
| Liver disease (n=5) | 5 | *C. bantiana* (3), *A. flavus* (2) | *-* |
| Trauma (n=1) | 1 | *A. fusispora (1)* | *-* |
| Steroid intake (n=21) | 17 | *C. bantiana* (9), *A. fumigatus (2), A. niger (1), A. flavus (1), A. fusispora (1), S. apiospermum (1)* | *A. flavus (1), A. fumigatus (1)* |
| Other immunosuppressants (n=16) | 13 | *C. bantiana (4), A. flavus (3), A. fumigatus (1), A. fusispora (1), S. apiospermum (1)* | *A. flavus (1), A. fumigatus (1), A. nidulans (1)* |
| Malignancy (n=15) | 12 | *A. flavus (5),*  *A. fumigatus (1), C. hawaiiensis (1)* | *A. fumigatus (2), A. flavus (1),*  *A. nidulans (1), A. fusispora (1)* |
| Renal transplant (n=7) | 7 | *C. bantiana (4), A. fusispora (1) A. fumigatus (1), S. apiospermum (1)* | *-* |
| Primary immunodeficiency (Chronic granulomatous disease) (n=2) | 2 | *-* | *A. fumigatus (1), A. nidulans (1)* |

***There is an overlap of cases in different risk factors.**

**Supplementary Table S2.** Clinico-mycological characteristics, treatment and outcome of adult and paediatric patients with CNS mucormycosis.

| Characteristic | Total (n=13) | Adults (n=11) | Paediatrics (n=2) |
| --- | --- | --- | --- |
| Age (mean ± SD) | 45 (23) | 46 (16) | 7 (3) |
| Females | 3 (23.1) | 1 (9.1) | 2 (100) |
| Males | 10 (76.9) | 10 (90.9) | 0 (0) |
| Symptoms |  |  |  |
| Duration of symptoms (days) (mean ± SD) | 21 (15) | 21 (15.5) | 21 (6) |
| Symptom duration < 5 days | 1 (7.7) | 1 (9.1) | 0 (0) |
| Symptom duration < 30 days | 9 (69.2) | 7 (63.6) | 2 (100) |
| Headache | 8 (61.5) | 8 (72.7) | 0 (0) |
| Fever | 5 (38.5) | 5 (45.5) | 0 (0) |
| Seizure | 3 (23.1) | 3 (27.3) | 0 (0) |
| Hemiparesis | 3 (23.1) | 3 (27.3) | 0 (0) |
| Altered sensorium | 3 (23.1) | 2 (18.2) | 0 (0) |
| Vomiting | 1 (7.7) | 0 (0) | 1 (50.0) |
| Risk factors |  |  |  |
| No identifiable risk (immunocompetent) | 5 (38.5) | 5 (38.5) | 0 (0) |
| Diabetes mellitus | 6 (46.2) | 5 (45.5) | 1 (50.0) |
| Steroid intake | 2 (15.4) | 1 (9.1) | 1 (50.0) |
| Trauma | 1 (7.7) | 1 (9.1) | 0(0) |
| Malignancy | 1 (7.7) | 0 (0) | 1 (50.0) |
| Immunosuppressants | 1 (7.7) | 0 (0) | 1 (50.0) |
| Part of brain involvement |  |  |  |
| Frontal lobe | 8 (61.5) | 7 (63.6) | 1 (50.0) |
| Parietal lobe | 3 (23.1) | 2 (18.2) | 1 (50.0) |
| Temporal lobe | 3 (23.1) | 3 (27.3) | 0 |
| Occipital lobe | 2 (15.4) | 1 (9.1) | 1 (50.0) |
| Other sites | 1 (7.7) | 1 (9.1) | 0 |
| Number of lesions |  |  |  |
| Single | 9 (69.2) | 8 (72.7) | 1 (50.0) |
| Multiple | 4 (30.8) | 3 (27.3) | 1 (50.0) |
| Microscopy (aseptate hyphae) | 13 (100) | 11 (100) | 2 (100) |
| Fungal isolate (culture positive) | 7 (53.8) | *R. arrhizus* (n=5), *R. microsporus* (n=2) | - |
| Medical therapy | 12 (92.3) | 10 (90.9) | 2 (100) |
| Liposomal amphotericin B | 11 (84.6) | 9 (81.8) | 2 (100) |
| Posaconazole | 1 (7.7) | 1 (9.1) | 0 (0) |
| Surgery | | | |
| Partial excision | 6 (46.2) | 5 (45.5) | 1 (50.0) |
| Complete excision | 7 (53.8) | 6 (54.5) | 1 (50.0) |
| Outcome | | | |
| Death | 5 (38.5) | 4 (36.4) | 1 (50) |

**Supplementary Table S3**: Comparison of clinical characteristics, treatment and outcome of patients with CNS IMI due to *Cladophialophora bantiana* vs *Aspergillus* spp*.*

| Characteristic | Total (80) | *Cladophialophora bantiana* (21) | *Aspergillus spp* (59) | Odds Ratio (95% Confidence interval) | P value |
| --- | --- | --- | --- | --- | --- |
| Age (mean ± SD) | 28 (24) | 34 (19.25) | 25 (26) | - | 0.53 |
| Paediatric | 16 (20.0) | 0 (0) | 16 (27.1) | **-** | **Ref** |
| Adult | 64 (80.0) | 21 (100) | 43 (72.9) | **-** | **0.008** |
| Females | 24 (30.0) | 4 (19.0) | 20 (33.9) | - | Ref |
| Males | 56 (70.0) | 17 (81.0) | 39 (66.1) | 0.459 (0.136-1.547) | 0.20 |
| Symptoms |  |  |  |  |  |
| Duration of symptoms (days) (mean ± SD) | 15 (23) | 11.5 (13) | 15 (23.5) |  | 0.12 |
| Symptom duration < 5 days | 18 (22.5) | 4 (19.0) | 14 (23.7) | 1.322 (0.381-4.585) | 0.66 |
| Headache | 45 (56.3) | 16 (76.2) | 29 (49.2) | 0.302 (0.098-0.932) | **0.03** |
| Seizure | 45 (56.3) | 14 (66.7) | 31 (52.5) | 0.554 (0.195-1.568) | 0.26 |
| Fever | 31 (38.8) | 13 (61.9) | 18 (30.5) | 0.270 (0.095-0.765) | **0.01** |
| Altered sensorium | 29 (36.3) | 10 (47.6) | 19 (32.2) | 0.523 (0.189-1.443) | 0.21 |
| Vomiting | 27 (33.8) | 9 (42.9) | 18 (30.5) | 0.585 (0.210-1.634) | 0.30 |
| Hemiparesis | 16 (20.0) | 7 (33.3) | 9 (15.3) | 0.360 (0.114-1.139) | 0.08 |
| Focal neurological deficit | 7 (8.8) | 1 (4.8) | 6 (10.2) | 2.264 (0.256-20.001) | 0.46 |
| Risk factors |  |  |  |  |  |
| No identifiable risk (immunocompetent) | 58 (77.9) | 14 (66.7) | 44 (74.6) | 1.467 (0.498-4.319) | 0.48 |
| Diabetes Mellitus | 6 (7.5) | 3 (14.3) | 3 (5.1) | 0.321 (0.060-1.735) | 0.18 |
| Kidney disease | 7 (8.8) | 6 (28.6) | 1 (1.7) | 0.043 (0.005-0.386) | **0.005** |
| Liver disease | 5 (6.3) | 3 (14.3) | 2 (3.4) | 0.211 (0.033-1.361) | 0.10 |
| Immunocompromised | 22 (27.5) | 7 (33.3) | 15 (25.4) | - | Ref |
| Steroid intake | 16 (20.0) | 9 (42.9) | 7 (11.9) | **0.179 (0.056-0.578)** | **0.004** |
| Immunosuppressants | 11 (13.8) | 4 (19.0) | 7 (11.9) | 0.572 (0.149-2.196) | 0.41 |
| Renal transplant | 5 (6.3) | 4 (19.0) | 1 (1.7) | 0.073 (0.008-0.700) | **0.023** |
| Part of brain involvement |  |  |  |  |  |
| Frontal lobe | 57 (71.3) | 12 (57.1) | 45 (76.3) | 1.903 (0.612-5.916) | 0.26 |
| Parietal lobe | 32 (40) | 10 (47.6) | 22 (37.3) | 0.333 (0.044-2.532) | 0.28 |
| Temporal lobe | 13 (16.3) | 2 (9.5) | 11 (16.3) | 2.177 (0.441-10.757) | 0.34 |
| Occipital lobe | 6 (7.5) | 3 (14.3) | 3 (5.1) | 0.321 (0.060-1.735) | 0.18 |
| Other sites | 16 (20.0) | 2 (9.5) | 14 (23.7) | 2.956 (0.611-14.287) | 0.17 |
| Number of lesions |  |  |  |  |  |
| Single | 53 (66.3) | 14 (66.7) | 39 (66.1) | - | Ref |
| Multiple | 27 (33.8) | 7 (33.3) | 20 (33.9) | 0.975 (0.339-2.801) | 0.962 |
| Medical treatment |  |  |  |  |  |
| Medical therapy | 73 (91.3) | 20 (95.2) | 53 (89.8) | 0.442 (0.050-3.902) | 0.46 |
| Voriconazole | 51 (63.7) | 13 (61.9) | 38 (64.4) | 1.114 (0.398-3.117) | 0.838 |
| Amphotericin B | 22 (27.5) | 8 (38.1) | 14 (23.7) | 0.506 (0.174-1.467) | 0.21 |
| Amphotericin B deoxycholate | 8 (10.0) | 4 (19.0) | 4 (6.8) | 0.309 (0.070-1.370) | 0.12 |
| Liposomal amphotericin B | 14 (17.5) | 4 (19.0) | 10 (16.9) | 0.867 (0.240-3.132) | 0.828 |
| Surgical management |  |  |  |  |  |
| Partial excision | 29 (36.3) | 10 (47.6) | 19 (32.2) | - | Ref |
| Complete excision | 51 (63.7) | 11 (52.4) | 40 (67.8) | 0.523 (0.189-1.443) | 0.21 |
| Outcome |  |  |  |  |  |
| Death | 24 (30.0) | 8 (38.1) | 16 (27.1) | 1.654 (0.578-4.732) | 0.34 |

**Supplementary figures**


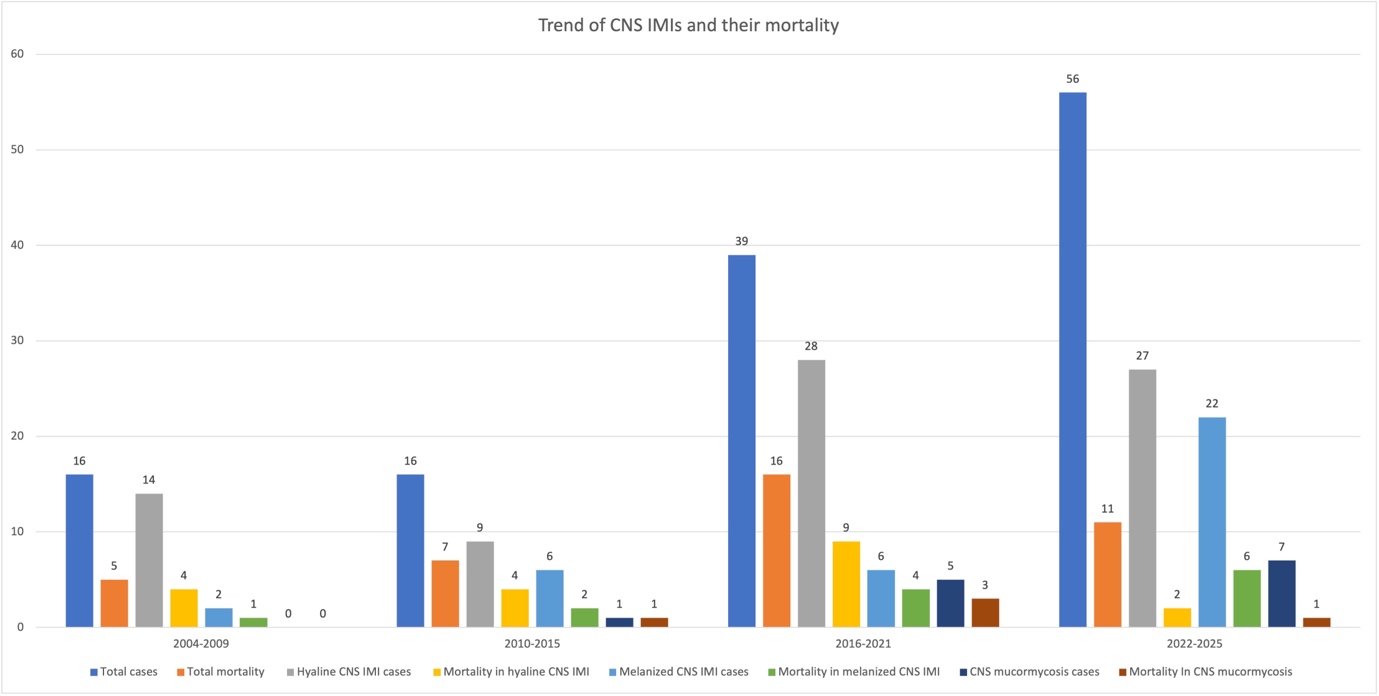


**Supplementary Fig. S1.** Six-yearly trends of the number of CNS IMI cases and mortality (2004-2025).

**
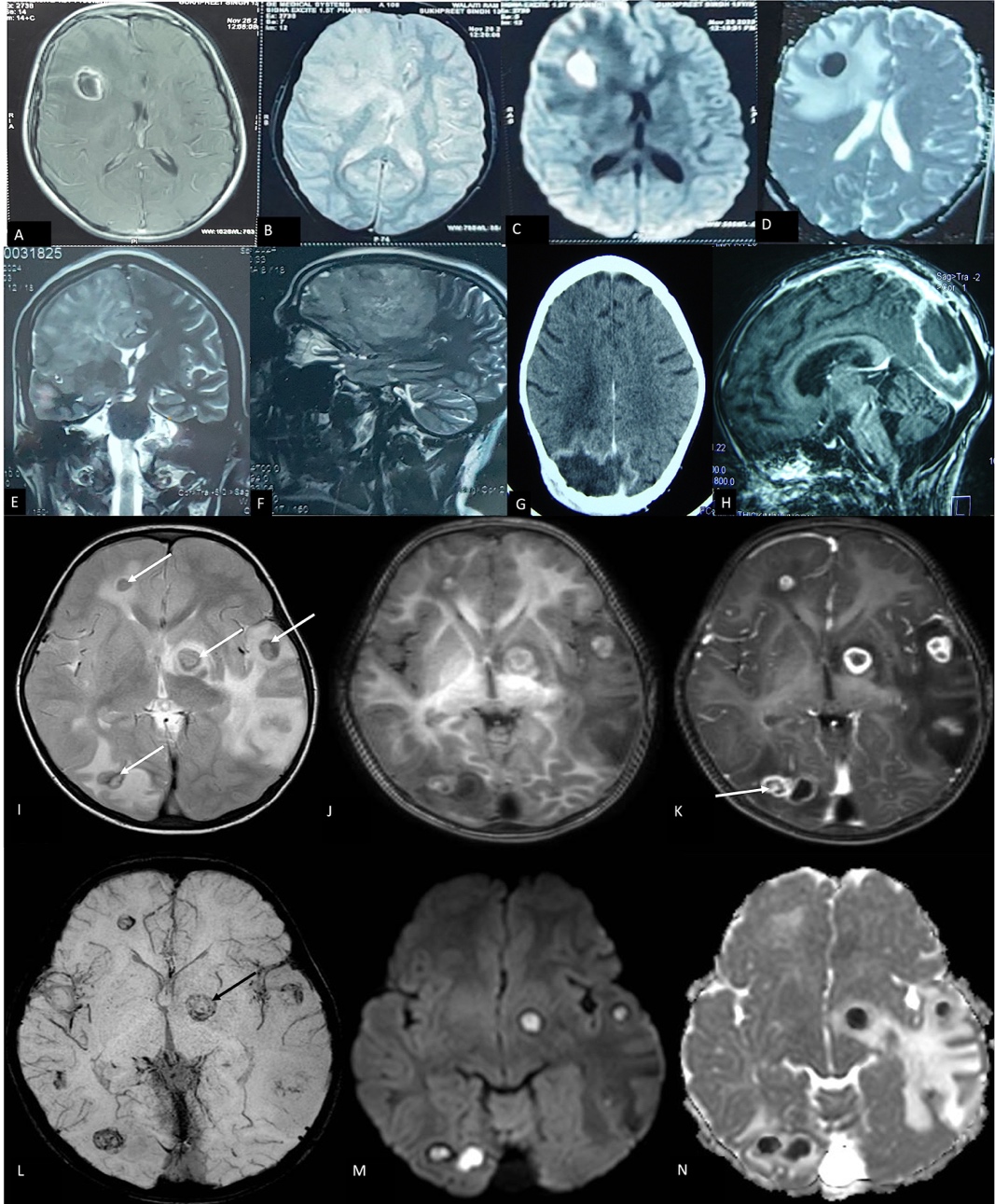
**

**Supplementary Fig. S2.** Radiological findings in the central nervous system invasive mould infections: (A-D) Radiological imaging in a 13-year-old immunocompetent male diagnosed with cerebral phaeohyphomycosis by *C. bantiana*. Axial post-contrast T1-weighted image (A) showing ring enhancing lesion (REL) in the right frontal lobe with surrounding edema and mild mass effect. Axial T2-gradient echo (GRE, B) image showing blooming in the wall (arrow) of the REL suggestive of hemorrhage. Diffusion weighted image (DWI, C) and apparent diffusion coefficient (ADC, D) images showing diffusion restriction (arrow) within the REL. (E-F) Radiological imaging in a 33-year-old immunocompetent male diagnosed with cerebral phaeohyphomycosis by *Chaetomium* spp. Coronal (E) and sagittal (F) T2-weighted images showing heterogeneously hyperintense lesion involving the right frontal lobe. It is causing mild mass effect in form of effacement of extra-axial spaces, compression of right lateral ventricle and mild contralateral midline shift. (G-H) Radiological imaging in a 4-year-old female suffering from ALL diagnosed with cerebral mucormycosis. Axial contrast-enhanced CT (G) and sagittal post-contrast T1-weighted (H) images showing low attenuation/intensity lesion with thick peripheral enhancement (arrows) in right parieto-occipital region. (I-N) Radiological imaging in a 3-year-old immunocompetent male diagnosed with multiple brain abscesses caused by *Aspergillus fumigatus.* Axial T2-(I), T1-weighted (J) and post-contrast T1-weighted (K) images showing multiple ring-enhancing lesions (white arrows) with variable perilesional edema in bilateral cerebral hemispheres.  A few of the lesions show internal projections (arrow in K). Susceptibility weighted image (SWI, L) showing hemorrhages in the wall. Diffusion weighted images (DWI, M) and apparent diffusion coefficient (ADC) map images (N) showing diffusion restriction.

**
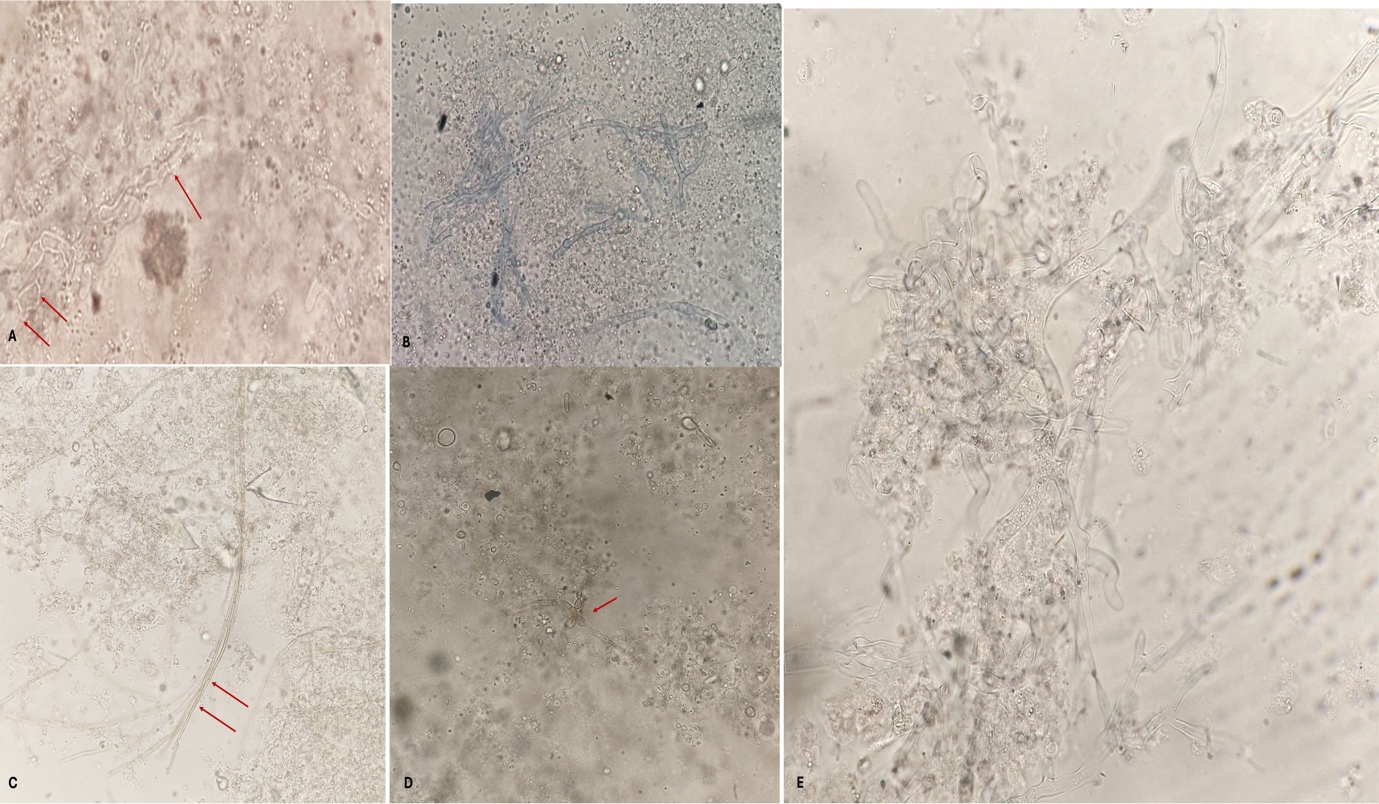
**

**Supplementary Fig. S3.** Microscopic findings of the 10% KOH mount (40X) from samples from CNS IMI cases showing A) hyaline septate hyphae in a 65-year-old immunocompetent male with multiple abscesses in frontal and temporal lobes which grew *A. fumigatus* (hyalohyphomycosis), B) hyaline septate hyphae in a 2-year-old female diagnosed with ALL having multiple abscesses in frontal lobe and parasagittal areas which grew *A. fumigatus* (hyalohyphomycosis), C) melanized thin septate hyphae in 17-year-old immunocompetent male with single abscess in frontal lobe which grew *C. bantiana* (phaeohyphomycosis), D) melanized thin septate hyphae in 41-year-old immunocompetent male with multiple abscesses in frontal lobe which grew *C. bantiana* (phaeohyphomycosis) E) broad aseptate hyphae in a 46-year-old male on steroids with multiple abscesses in frontal lobe (mucormycosis).

**
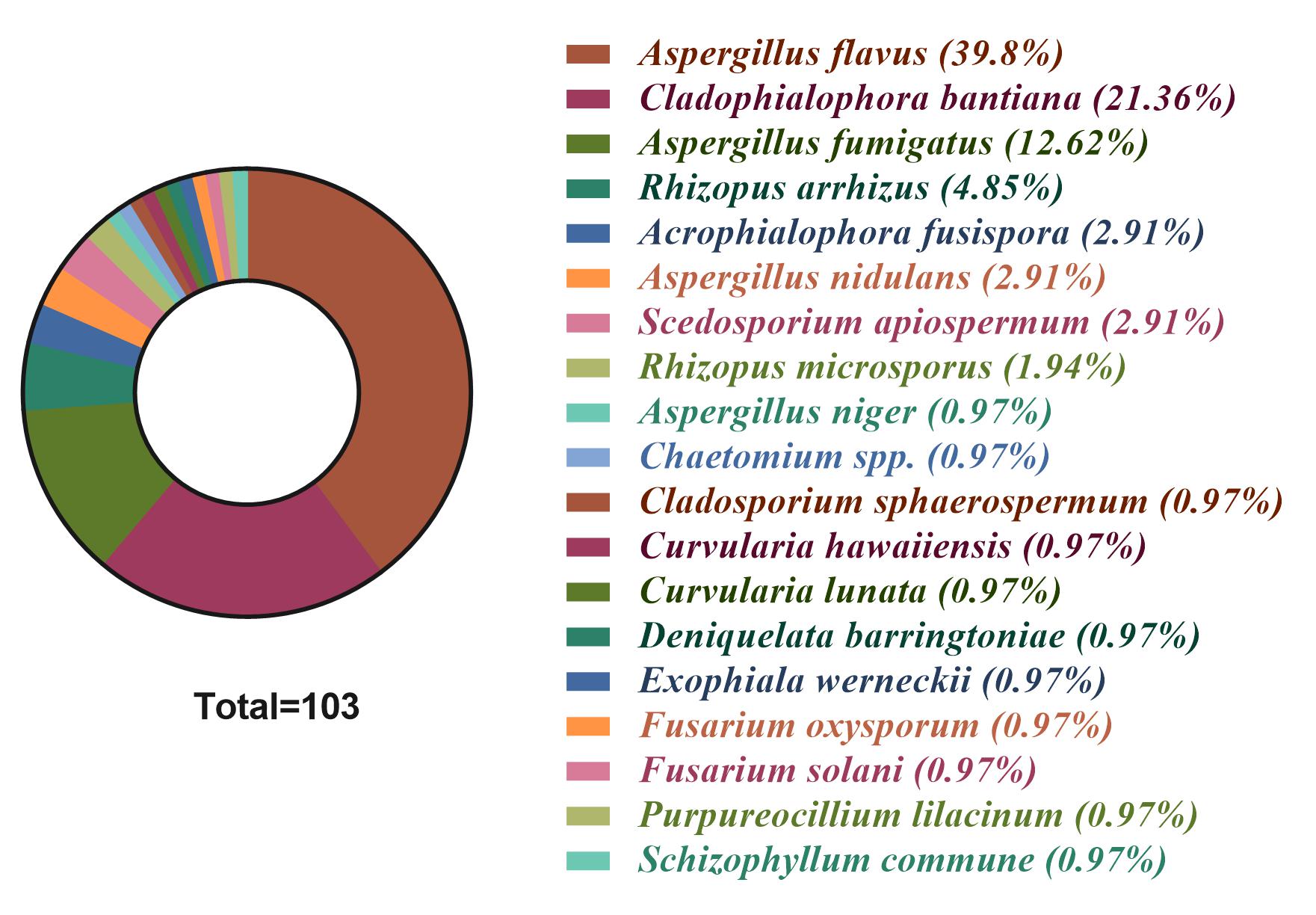
**

**Supplementary Fig. S4.** Spectrum of fungal pathogens isolated from central nervous system invasive mould infection cases.

**
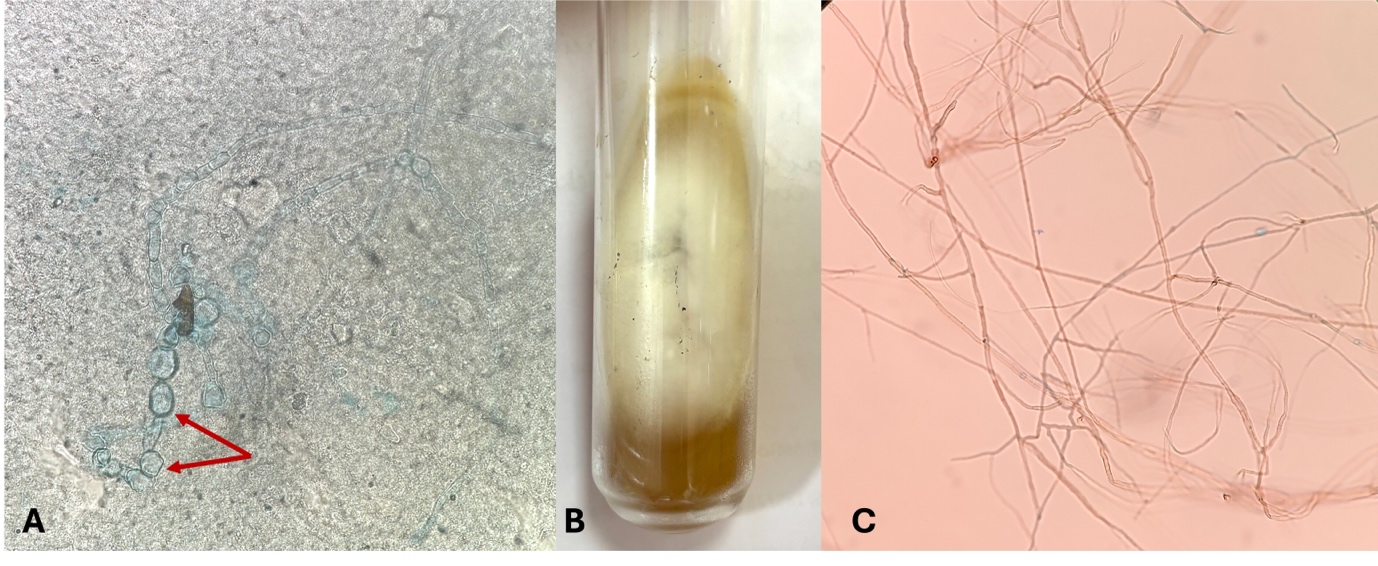
**

**Supplementary Fig. S5.** A)10% KOH mount from temporal brain abscess of a 65-year-old immunocompetent male showing melanized septate hyphae with bulbous swellings (soft pointer towards melanized fungi) (40X). B) Culture on Sabouraud dextrose agar showing creamish yellow colonies of *Deniquelata barringtoniae*. C) The lactophenol cotton blue mount showed melanized septate hyphae without sporulation (40X).


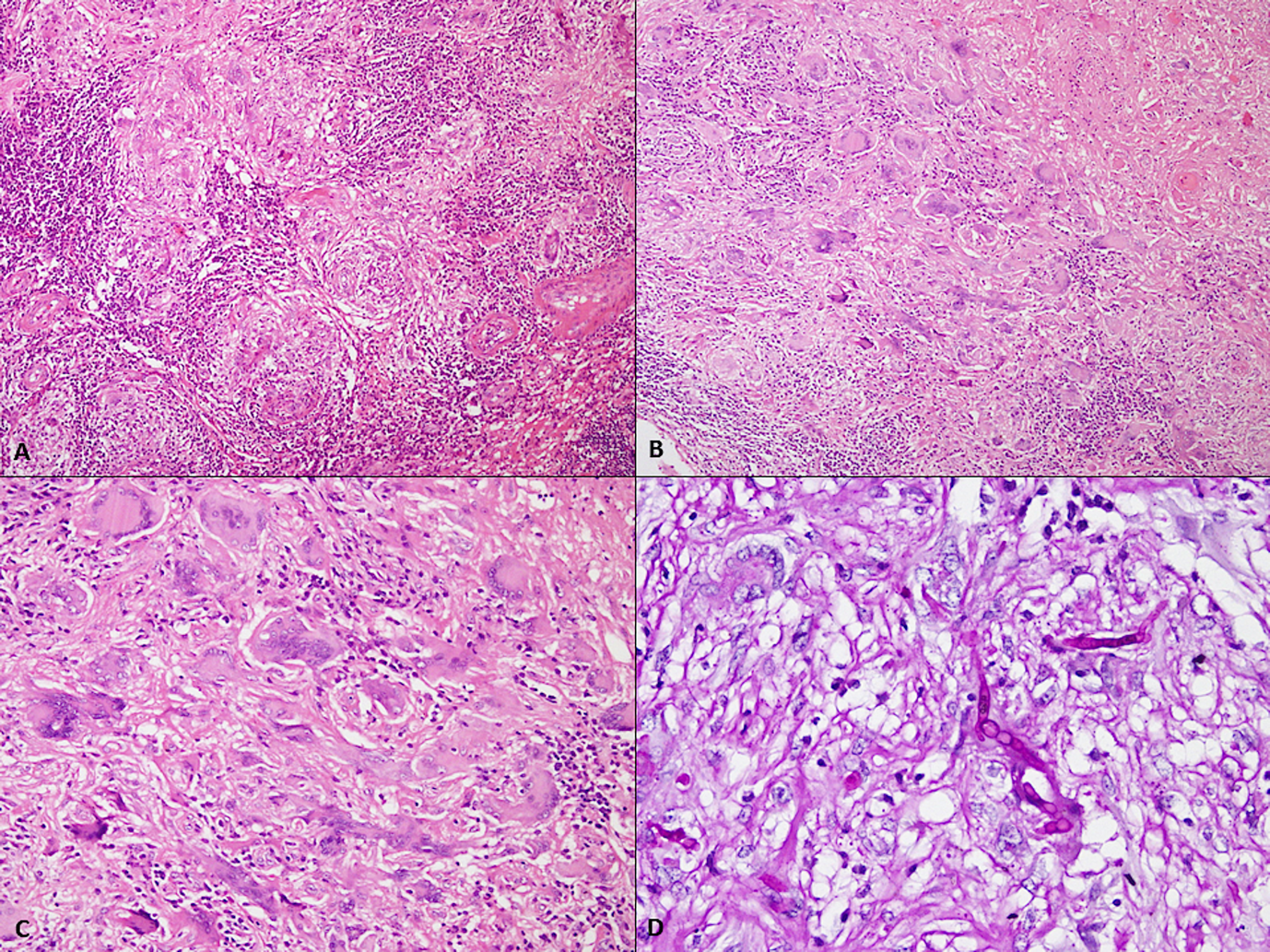


**Supplementary Fig. S6** (A) Low magnification depicting numerous well-formed granuloma in a background of dense sclerosis (H&E, 10X); B) Ill-formed granulomas with loosely scattered giant cells with necrosis in the surrounding areas (H&E, 10X); C) High magnification depicting scattered giant cells containing negative shadows of fungal hyphae (H&E, 40X); D) Periodic-acid Schiff (PAS) stain highlighting slender, septate hyphae (H&E, 40X).


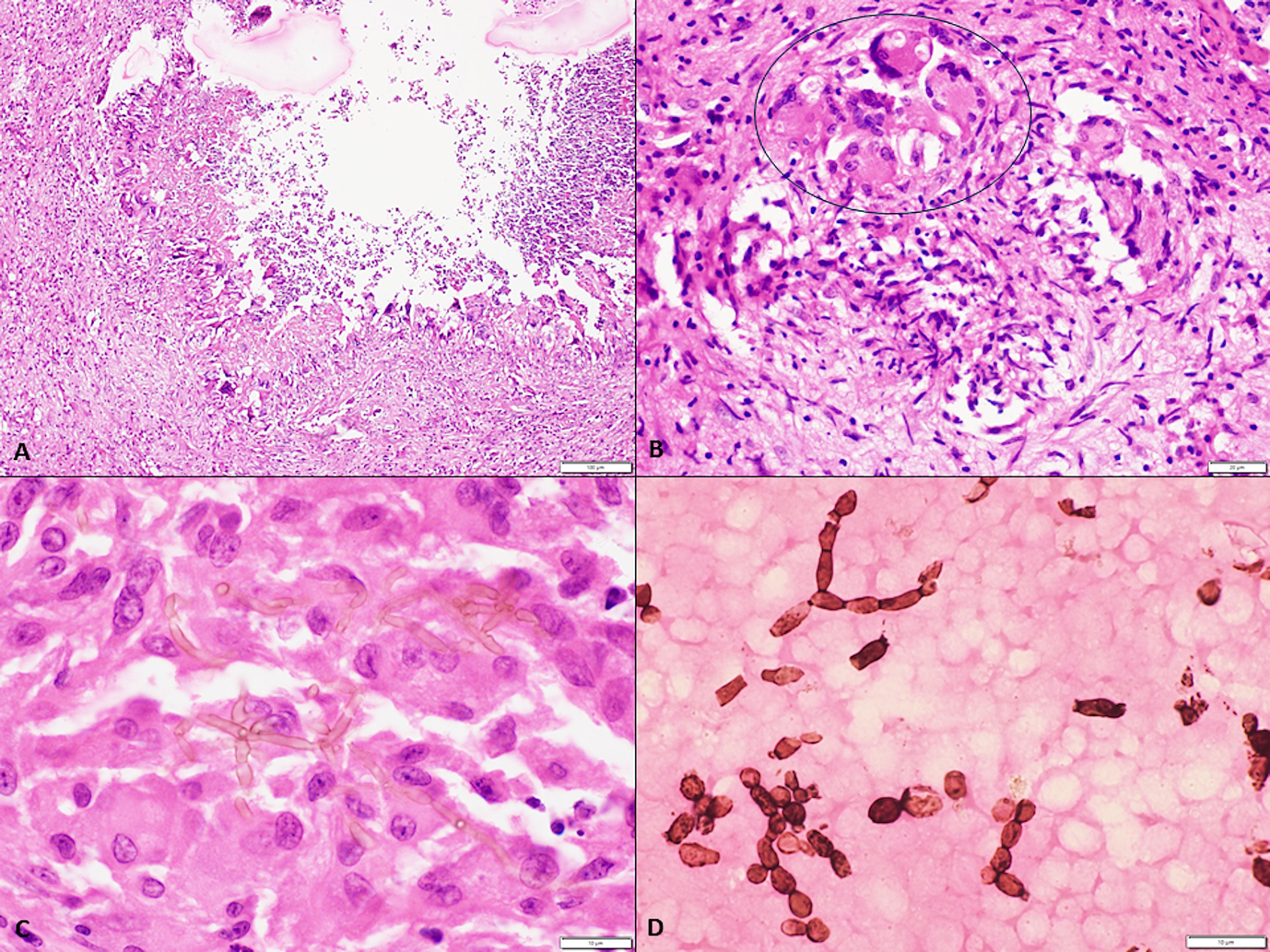


**Supplementary Fig. S7.** Low magnification depicting A) Central cystic necrosis bordered by a fibrotic wall with palisading histiocytes (H&E, 10X); B) Granulomatous reaction (encircled) with giant cells containing negative shadows. Many scattered histiocytes and lymphocytes are seen in the background (H&E, 40X); C) Thin branching melanized hyphae with prominent septate constrictions (H&E, 100X); D) Masson Fontana stain highlighting the melanin-producing fungi (H&E, 100X).


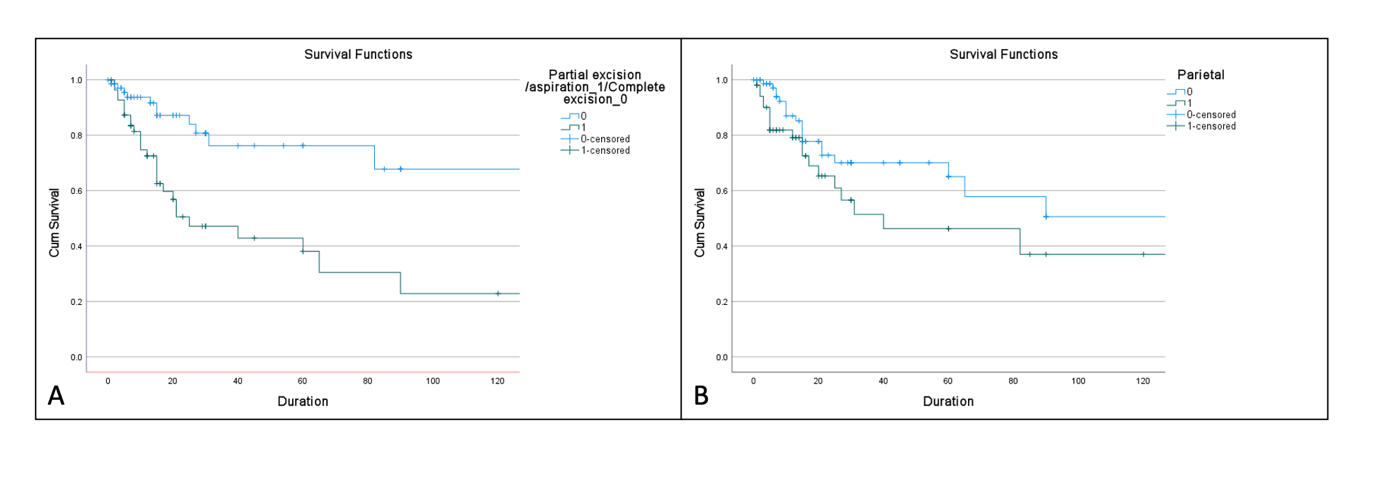


**Supplementary Fig. S8**. Kaplan–Meier survival curves depicting comparative cumulative survival in (A) patients treated with partial excision (green) and complete excision (blue) (p=0.02), (B) patients with lesions in the parietal lobe (green) and lesions in other lobes of the brain (blue) (p=0.048).


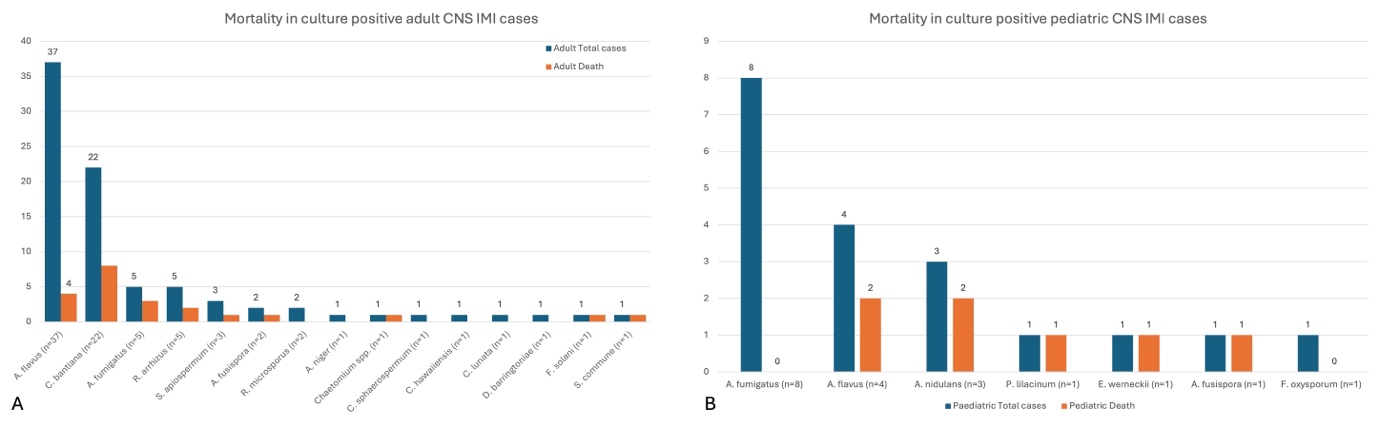


**Supplementary Fig. S9:** Species wise distribution and mortality of CNS IMI patients in adults and paediatrics


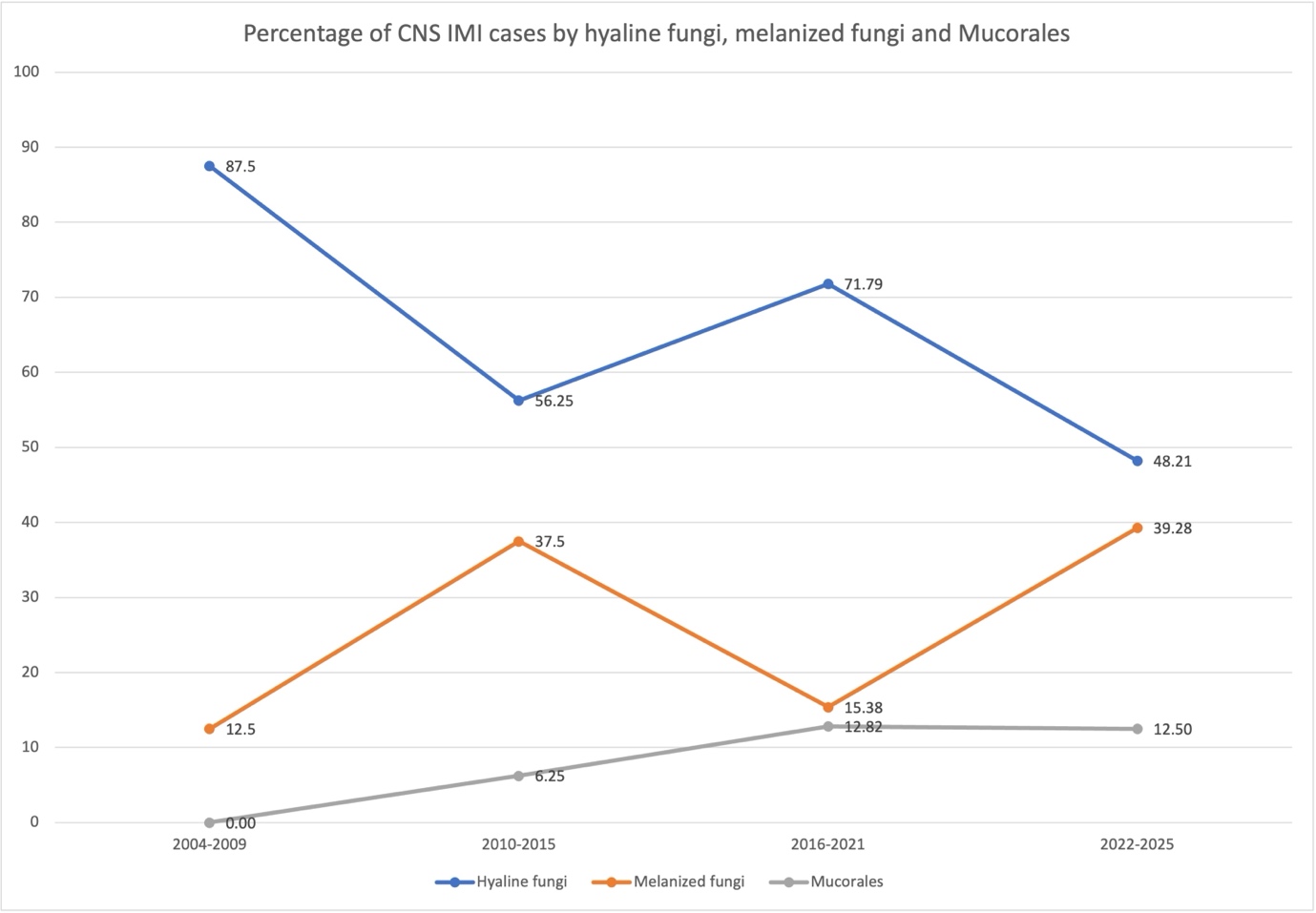


**Supplementary Fig. S10:** Percentage of CNS IMI patients by hyaline fungi, melanized fungi and Mucorales

**
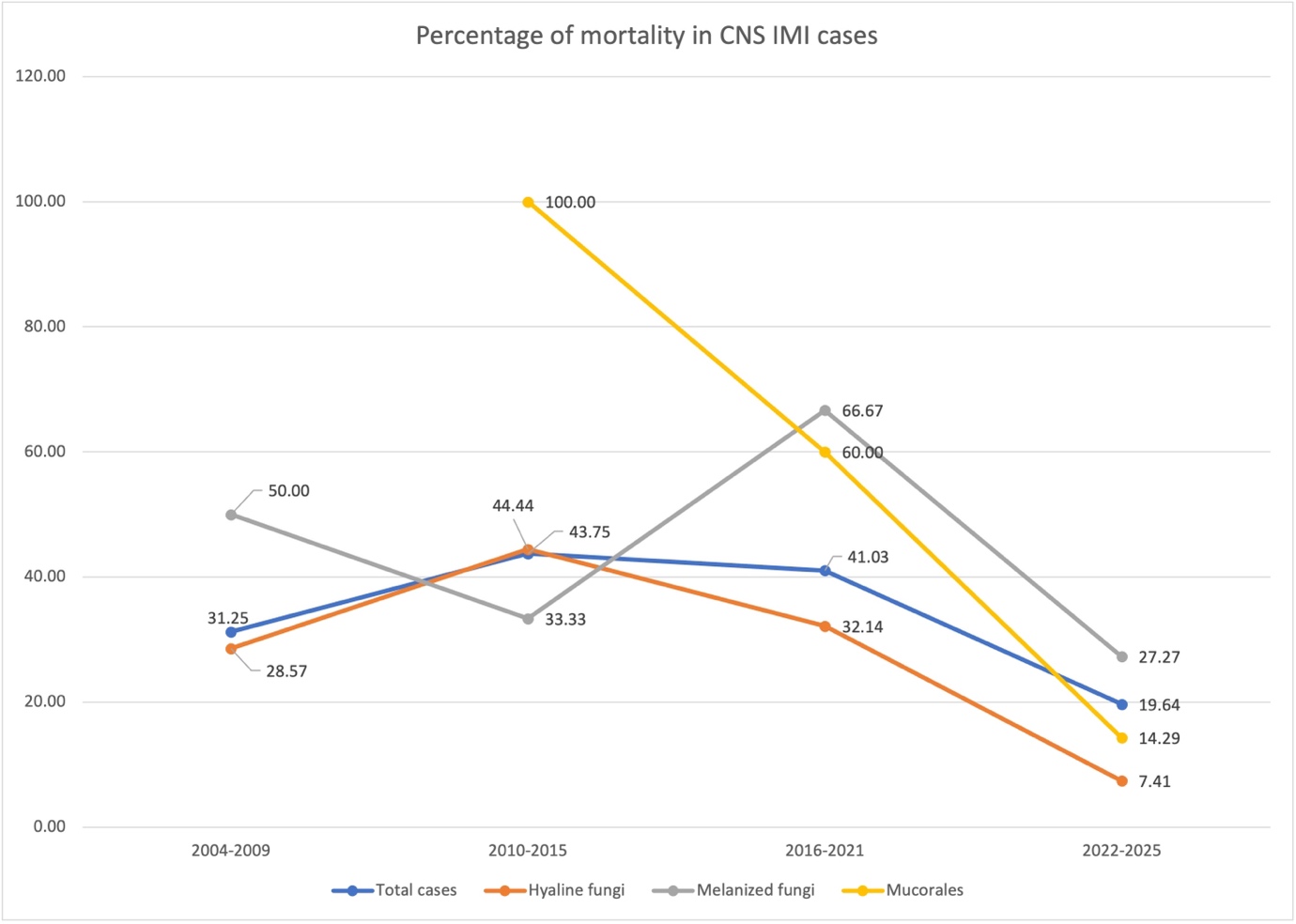
**

**Supplementary Fig. S11:** Percentage of mortality in CNS IMI patients by hyaline fungi, melanized fungi and Mucorales
